# Insights into Infusion-Based Targeted Drug Delivery in the Brain: Perspectives, Challenges and Opportunities

**DOI:** 10.3390/ijms23063139

**Published:** 2022-03-15

**Authors:** Asad Jamal, Tian Yuan, Stefano Galvan, Antonella Castellano, Marco Riva, Riccardo Secoli, Andrea Falini, Lorenzo Bello, Ferdinando Rodriguez y Baena, Daniele Dini

**Affiliations:** 1 Department of Mechanical Engineering, Imperial College London, London SW7 2AZ, UK; t.yuan19@imperial.ac.uk (T.Y.); s.galvan@imperial.ac.uk (S.G.); r.secoli@imperial.ac.uk (R.S.); f.rodriguez@imperial.ac.uk (F.R.y.B.); 2 Vita-Salute San Raffaele University, 20132 Milan, Italy; castellano.antonella@hsr.it (A.C.); falini.andrea@hsr.it (A.F.); 3 Neuroradiology Unit and CERMAC, IRCCS Ospedale San Raffaele, 20132 Milan, Italy; 4 Department of Medical Biotechnology and Translational Medicine, Università degli Studi di Milano, Via Festa del Perdono 7, 20122 Milan, Italy; marco.riva@hunimed.eu; 5 Department of Oncology and Hematology-Oncology, Universitá degli Studi di Milano, 20122 Milan, Italy; lorenzo.bello@unimi.it

**Keywords:** convection-enhanced delivery, brain, infusion, fluid flow, mass transport, tissue deformation, molecular interactions

## Abstract

Targeted drug delivery in the brain is instrumental in the treatment of lethal brain diseases, such as glioblastoma multiforme, the most aggressive primary central nervous system tumour in adults. Infusion-based drug delivery techniques, which directly administer to the tissue for local treatment, as in convection-enhanced delivery (CED), provide an important opportunity; however, poor understanding of the pressure-driven drug transport mechanisms in the brain has hindered its ultimate success in clinical applications. In this review, we focus on the biomechanical and biochemical aspects of infusion-based targeted drug delivery in the brain and look into the underlying molecular level mechanisms. We discuss recent advances and challenges in the complementary field of medical robotics and its use in targeted drug delivery in the brain. A critical overview of current research in these areas and their clinical implications is provided. This review delivers new ideas and perspectives for further studies of targeted drug delivery in the brain.

## 1. Introduction

Tumours in the central nervous system (CNS) are some of the most prevalent, lethal and yet poorly treated diseases within the brain. Glioblastoma multiforme (GBM), a grade IV glioma, is the fastest-growing and most aggressive malignant primary CNS tumour in adults. It primarily occurs in older patients, with an average age of 64 years at diagnosis. Survival rates are poor, with approximately 40% survival in the first year post diagnosis and 17% in the second year. GBMs lead to about 250,000 deaths per year worldwide, and their treatment cost in Europe in 2010 was about 5.2 billion euros [1,2,3]. Conventional techniques such as chemotherapy and radiation are not effective in treating GBM. They either suffer from limitations in passing drugs through the blood–brain barrier (BBB) or unwanted drug distribution throughout the tissue due to passive diffusion and poor delivery to the target, or they are not viable because of severe side effects, e.g., localised tissue damage [4,5,6]. The drug effects within the CNS are driven by the concentration–time profile at the target site, and therefore, drugs need to reach the target for as long as needed and in an appropriate concentration, neither of which is easily achievable with conventional diffusion-based delivery methods. To overcome these challenges, an emerging approach is infusion-based targeted drug delivery, such as convection-enhanced delivery (CED), performed with robotic steerable needles [7,8].

Recent advancements in medical robotics through technical innovations has led to significant improvements in CED-like technologies [9,10]; however, ultimate success in the clinical applications of these systems remains a goal to be accomplished [7]. Current embodiments suffer from a lack of precise information and reliable experimental data on the flow behaviour in the brain, which limits the development of precise numerical models and their implementation in automated surgical systems. In fact, to progress towards a reliable and automated system for infusion-based targeted drug delivery to the brain, advancements on four fronts are to be made: (1) characterisation and understanding of drug flow behaviour in complex brain tissue, (2) developing realistic models based on experimental data that can predict drug infusion at the pre-operative stage, (3) technical innovation in drug delivery tools, and (4) their clinical deployment in Randomised Controlled Trials (RCTs). Please note this paper focus on GBM as a relevant example, but the strategies can also be used for treating other pathologies in the brain including Parkinson’s disease and Alzheimer’s disease [7,11].

This review aims to summarise the concepts, recent advances and limitations in brain tissue characterisation for applications in CED-like technologies, modelling the infusion-based drug flow in complex anisotropic brain tissue and the use of specific drugs for the treatment of lethal brain tumours. Furthermore, we discuss the current state of the art of robotic steerable needles for drug delivery in the brain and their clinical implications.

## 2. Brain Tissue: A Complex System for Diffusion-Based Drug Delivery

The brain is a biological system mainly composed of neurons and neuroglia and possesses extreme complexity arising from the interaction of about 86 billion neurons and 100 trillion connections [12]. A prominent obstacle to the drug transport inside the brain is the BBB, which separates blood from the brain. The BBB, primarily formed by brain capillary endothelial cells connected by tight junctions that constitute the walls of the brain capillaries, is a selective barrier that tightly regulates the movement of ions, molecules and cells between the blood and the CNS. Properties of drug such as molecular weight and affinity for a lipid environment affect their ability to pass BBB. The BBB allows small molecules to pass through but not macromolecules [13,14]. In addition, transport of even small drug molecules across the BBB is affected by helper molecules that move drugs from the blood to the brain. The drug may bind to targeted binding sites and to other tissue components that should be non-binding sites, affecting the final concentration–time profile of the drug at target site, which determines the pharmacodynamic effect over time [15]. Inside the brain, several factors can influence drug distribution, e.g., bulk flow of extracellular fluid (ECF), cerebrospinal fluid (CSF) and extra-cellular exchange. Furthermore, once having crossed the BBB, drug distribution within the ECF is also affected by the tortuosity of the tissue, leading to a relatively smaller effective diffusion [16,17].

Specially, the CNS tissue as an anisotropic composite material is a complex system for drug flow and distribution. Biomechanically, it can be broadly characterised by the stiff directional axons wrapped in insulating lipid-rich layers (myelin) that are supported by a soft matrix composed of glial cells and a network of biopolymers, the extracellular matrix (ECM). The directional axons can be a mechanical obstacle to drug diffusion and spatial distribution in the CNS tissue [18,19]. The presence of elongated axons can compel the drug particles to diffuse around fibre-like obstructions, hence the increased tortuosity and reduced effective diffusion. Furthermore, inside the tissue, it is the ECM that regulates the local transport of molecules. It provides selective filtering for nanoparticles (NPs) through its interactions. The filtering properties of the ECM can be deleterious for diffusion-based drug delivery and could perturb their flow behaviour and spatial distribution [20,21,22]. For example, it has been shown that presence of surface charge on particles can significantly suppress their diffusion in the ECM [23]. The role of ECM in drug flow is further discussed in detail in Section 3.2.

In brief, drug transport by diffusion often suffers from the loss of macromolecules across the BBB, binding to the receptors and uptake into cells. This issue complicates brain disease treatment by diffusion-based drug delivery therapies; therefore, no promising results have been achieved.

## 3. Infusion-Based Drug Delivery in CNS Tissue

**Figure 1:**
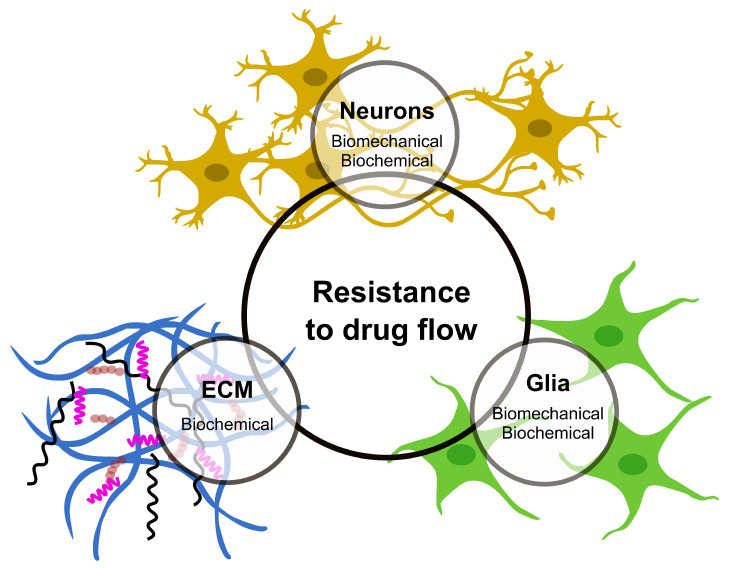
The CNS components that offer major resistance to drug flow and distribution.

Infusion-based transport provides an opportunity to overcome most of the challenges faced by drug delivery via diffusion in the CNS tissue. In contrast to the diffusion-based approach, which relies on concentration gradients, a positive pressure gradient drives the flow to the targeted area, also known as convective transport. There is a growing interest in understanding the pressure-driven drug delivery and underlying mechanisms for applications in CED-like technologies; however, challenges still exist. The main challenges include the unexpected relationship between drug distribution patterns and infusion parameters (such as the infusion rate, infusion volume and catheter angle), as well as backflow development, tissue edema and disruption of active tissue/BBB [7]. These, together with additional complexities due to brain tissue characteristics such as anisotropy and heterogeneity, lead to unexpected drug distribution patterns [24]. Progress mostly suffers from the lack of precise information and experimental data on how drug flow in CNS tissue is affected by its components (Figure 1), which could provide a fundamental mechanism to develop predictive models for drug flow and distribution. This problem is two-fold: (1) biomechanical aspects of infusion-based transport, i.e., understanding of flow field in CNS tissue and its corresponding mechanical response and (2) the molecular process involved between the drug and tissue components that influence drug flow and distribution. In this section, we will look into experimental studies focused on these aspects of infusion and discuss how a detailed knowledge of these processes, once developed, would eventually lead to predicting the drug flow and distribution in CNS tissue.

### 3.1. Biomechanical Aspects of Infusion-Based Drug Flow

Brain tissue is a soft porous composite material. When characterising its mechanical behaviour, earlier studies treated the tissue as a single-phase viscoelastic material [25,26,27,28,29,30] and ignored the influence due to possible relative motion of fluid through the tissue’s solid constituents. However, the explorative work of Franceschini et al. [31] showed that brain tissue exhibits consolidation-type behaviour in quasi-static deformation, i.e., pore flow deforms the solid matrix and fluid drainages from interstitial space. This, together with other advances in revealing the importance of fluid phase in physiological activities and evidence in the mechanical response of the tissue [32], has dictated the growing consensus of treating this soft tissue as a biphasic continuum, where a porous solid matrix is saturated with interstitial fluid [8,33,34,35]. It has been shown that infusion pressure can deform the CNS tissue and perturb the fluid flow behaviour [18]. Therefore, the localised mechanical properties of CNS have a key role in determining the pressure-driven flow behaviour and knowledge of both porous and viscous characteristics, such as hydraulic permeability, and the stiffness and compressibility of solid matrix is essential, e.g., for developing computational models that predict drug transport and interstitial flow in CNS. The existing experimental findings of such parameters at relevant length scales are rare, and results from computational models are often dependent on a number of assumptions which are difficult to verify, e.g., the constitutive parameters used in theoretical models of hydraulic permeability vary by up to three orders of magnitude [35,36,37,38,39,40].

Traditionally, the mechanics of brain tissue have been investigated using a range of different experimental techniques that measure the relevant properties at different length and time scales as well as different boundary and drainage conditions [31,41,42,43,44,45,46,47]. This results in discrepancies in experimentally observed parameters. For instance, the hydraulic permeability of biological specimens can be determined by compression [48,49], perfusion, i.e., drug delivery from a source with cross sectional surface comparable to the tissue dimensions [50,51,52] and infusion, i.e., localised drug delivery to a tissue from a point source (catheter’s tip) [52,53,54]. Initial experimental studies employed perfusion [37] and compression [31] approaches to determine brain hydraulic permeability; however, they did not consider the localised tissue heterogeneities due to the large size of the samples employed in their investigation. Moreover, the adopted perfusion set up in [37] is not compatible with infusion-based drug delivery methods. Therefore, the measured values cannot be used in developing models for localised drug delivery. Similarly, Greiner et al. [47] investigated the hydraulic permeability of human brain tissue using a compression approach; however, at the scales captured using large size samples, they also ignored the anisotropy of white matter, which is acceptable when studying the homogenised response of the tissue to deformation in the range of a few millimeters. This is not the case when localised processed are invoked, which are dominated by the tissue architecture in the sub-millimeter range. It is worth noting here that the anisotropy of the CNS tissue due to the directionality of the axons’ bundles [55,56] is an important factor to consider not only in drug infusion but also in its distribution after infusion and is critical in determining the efficacy of CED [57]. In contrast to compression and perfusion approaches, the infusion-based approach employed in our previous study to determine localised hydraulic permeability provided accurate quantitative information including the effect of directionality of axons on fluid flow. The hydraulic permeability was ∼65% lower when the flow was perpendicular to the axons than when the flow was parallel to the axons. The dependence of tissue hydraulic permeability on microstructural anisotropy has also been appreciated in previous computational findings [57].

Furthermore, microstructural heterogeneities among different regions of CNS tissue can affect the flow behaviour and distribution, though little is known about the detailed microstructure of CNS tissue. Only a recent study on fixed tissue of sheep brain has revealed localised microstructural differences such as axon volume fractions and geometry in specific regions, i.e., the corpus callosum, corona radiata and fornix of CNS [58]. Appreciating this fact, Vidotto et al. [19] used a computational approach and found a significant difference between hydraulic permeability in the corpus callosum and fornix. Using 3D reconstructed microstructure from electron microscopy images, they computed the corresponding permeability considering the tissue structure is mainly composed of directional axons. This is a significant finding; however, it is yet to be complemented by experimental findings. Additionally, experimental studies, which have looked in the different regions of CNS tissue and investigated the localised infusion mechanisms, are lacking.

During the infusion, CNS tissue is locally subjected to hydraulic pressure. This can lead to microstructural deformation that can affect the hydraulic permeability. For example, our recent study has revealed that the CNS tissue’s hydraulic permeability nonlinearly changes with infusion pressure [18]. Several factors can influence CNS deformation including infusate volume, tissue characteristics such as anisotropy or pre-existing conditions, e.g., edema due to hydrocephalus. The edema can also form due to infusion when the interstitial fluid (IF) volume increases and the low hydraulic permeability does not allow rapid distribution of the fluid. Edema refers to the condition where IF volume expansion or increased water content above the normal level (e.g., in hydrocephalus) produces swelling. This disturbance of solid and fluid volume balance affect drug flow and distribution, and the extent depends on the compliance of the CNS components [59,60]. It is important to note that due to anisotropy, the preferred deformations in CNS would be axon bundles being spread apart and not elongating [18]. Such microscopic expansion in CNS can also contribute to macroscopic deformation and cause the additional issue of backflow, i.e., the drug flowing back at the tissue–catheter interface.

Localised deformations can affect flow behaviour; however, detailed knowledge is yet to be developed to address existing limitations. Traditionally, Darcy’s law has been used to characterise the resistance to flow in a porous medium. The same is widely applied to quantify the hydraulic permeability of tissues, including CNS [18]. However, in the case of deformation in CNS tissue, the microstructure changes and therefore Darcy’s law needs to be modified to account for changing microstructure. It is not clear how the CNS microstructure changes because of infusion pressure and what is the relation between infusion pressure and deformation at the CNS components scale. For example, considering axons and ECM, the factors include whether axons are compressed, deformed or dislocated in ECM with respect to other accessory cells.

Another important biomechanical aspect of infusion-based flow is the tissue response to applied pressure. It is crucial to understand the mechanical behaviour of CNS tissue to understand, e.g., localised deformation at the tissue components scale. The mechanical characteristics of CNS tissue have been widely investigated using both macro and microscale experimental approaches [33]. Most of such characterisation is focused on the viscoelastic solid phase [61]; however, experimental investigations at the relevant length scale were able to capture the poroelastic effect as well [31,47,62]. Such experimental results are explained by treating brain tissue as a biphasic material. The basic biphasic theory assumes that under compressive loading, the relative motion between elastic solid phase and inviscid fluid phase develops a dissipation that governs the overall viscoelastic behaviour of the soft, porous tissue.

The basic biphasic model of soft tissues initially introduced by Mow et al. [63] for cartilage has been extended to integrate the non-linear elasticity of the solid phase under finite deformation and strain-dependent hydraulic permeability [64,65]. The experimental findings of flow-independent viscoelastic characteristics in cartilage led to the further addition of intrinsic viscoelasticity of the solid phase to the biphasic model [66,67], the so-called poro-viscoelastic (PVE) model. Such biphasic models have been applied to experimental results of mechanical testing on brain tissues in order to understand the mechanical characteristics [62,68]. Further details on the modelling of brain tissue can be found in Section 4.

Traditionally, the biphasic analysis of PVE behaviour of biological tissue considers a mechanically homogeneous porous solid phase and an interstitial fluid [69]. Cheng et al. [70] discussed the PVE behaviour of WM from macroscale stress relaxation experiments in unconfined compression without considering the consequences of localised mechanical heterogeneities of solid phase. Theoretical models such as those of Mehrabian et al. [71,72], which were developed to understand the PVE behaviour of macroscale experimental hydrocephalus data of brain tissue, also do not consider micromechanical details. However, solid-phase CNS tissue possesses micromechanical heterogeneities due to the presence of distinct tissue components, e.g., axons and ECM. Previous studies on isolated axons and ECM have reported several orders difference in their stiffness (Young’s modulus E of an isolated axon is ∼9.5 kPa [73] and only a few hundred Pascal of ECM [74]). Additionally, our recent study has revealed this difference when these components are in their natural environment. The stiffness of ECM is lower than that of axons, and the E of ECM when compared to axons is ∼47%, ∼42% and ∼25.6% lower in corpus callosum, corona radiata and fornix, respectively [61]. Furthermore, the existing trend in the literature can also be attributed to the absence of evidence that heterogeneities in the micromechanical environment affect flow behaviour. However, this has been recently provided by our experimental [18] and computational studies [19]. The emerging evidence of the microstructurally driven heterogeneous response of brain white matter to infusion pressure due to the deformability of axons and ECM strengthens the idea of modifying the traditional Darcy’s law and adopting a multiscale approach [75].

This progress in the biomechanical characterisation of CNS tissue for drug delivery applications is encouraging; however, this also clearly highlights the remaining unsolved problems. In particular, more experiments at a relevant length scale are needed to characterise CNS tissue and generate precise data to address questions such as the experimental determination of the permeability of different regions of CNS tissue, the pressure-dependence of hydraulic permeability, the localised deformation and edema formation with infusion pressure, and the mechanical behaviour of CNS components as well as the way they respond to pressure.

### 3.2. Chemical Aspects of Infusion-Based Drug Flow: Molecular-Level Mechanisms

**Figure 2:**
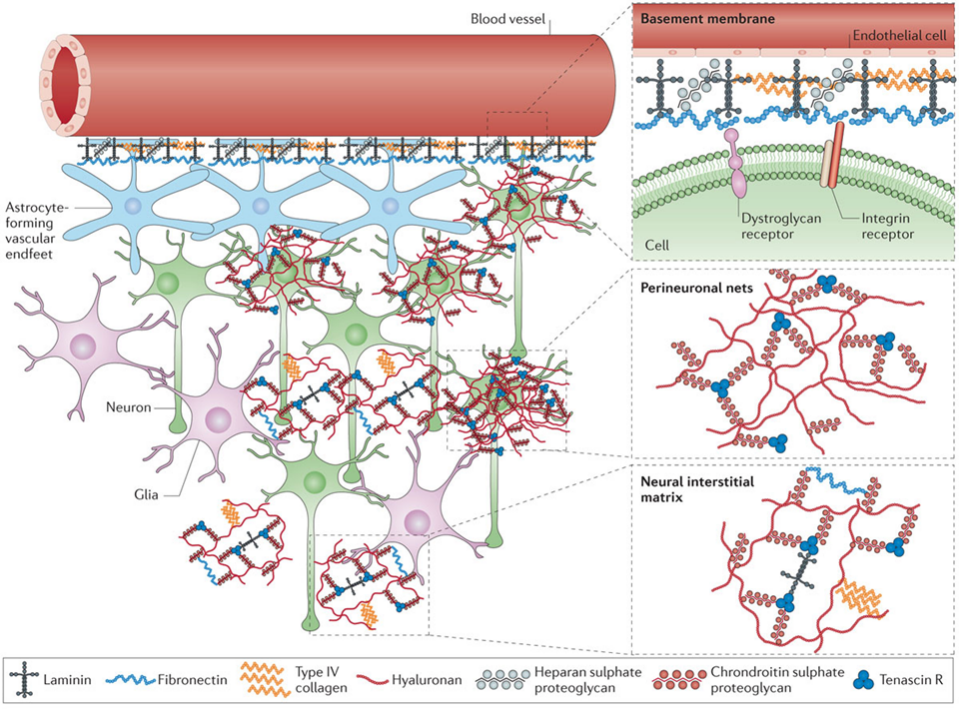
Extracellular matrix (ECM) components are arranged into basement membranes that lie outside cerebral vessels, condensed as perineuronal nets around the cell bodies and dendrites of neurons or diffusely distributed as the neural interstitial matrix between cells of the CNS parenchyma. The pink glial cells depict astrocytes, oligodendrocytes or microglia. This figure has been reproduced from [80] with permission from Springer Nature.

The current research is focused on the biomechanical aspects of infusion-based drug delivery; however, the chemical processes involved among the drug molecules and targeted tissue components as well as their surroundings have a crucial role and must not be ignored. In particular, the ECM in CNS is composed of hundreds of different biomolecules, mostly proteins and glycans (carbohydrate-based polymers made by all living organisms)—see Figure 2—that are covalently bonded in different configurations [76]. The ECM components, i.e., collagens, fibronectin, integrins, elastin and microfibrillar proteins, have explicit physiochemical properties and fulfil specific biological functions through various chemical interactions. These interactions act in highly complex and organised ways and drive processes including regulating interstitial fluid transport by selective filtering [21,22,77]. The presence of these molecules, chemically speaking a source of various interactions, also influences and perturbs the transport and distribution of external molecules or drug particles that try to traverse and reach, e.g., cancer cells. It has been observed that in the presence of cancer cells in CNS, the ECM relative volume and component density in comparison to other cells of the tissue matrix significantly increases. In gliomas, the relative volume of ECM increases from about 20% [78] (normal brain) to 48% [79]. The increased density of ECM components and larger relative volume in cancer tissue consequently increase the probability that drug particles will come across an increased number of biomolecules and their chemical interactions. This in turn can block the penetration of particles and affect the uniform delivery of drug particles to the targeted area in sufficient quantity [20,21].

Experimental investigations of particle motion in ECM have already started to hint at how much this complex network can influence the flow and distribution mechanisms. It is being recognised that NP interaction with various components of the ECM depends on the inherent properties of these components [81]. An important physiochemical parameter in this regard is the surface charge of macromolecules or particles that are transported through ECM. Charge particles or macromolecules electrostatically attract or repel charged components of the ECM that in return perturb their transport. In terms of electrostatics, the ECM is a network of unspecific localised charged patches through which the ECM provides selective filtering for the charged particles’ motion and regulates the transport of local molecules [82]. For example, collagen fibres possess a slightly positive charge in neutral pH [83,84] that can push them to interact with negatively charged particles and form aggregates. On the other hand, glycosaminoglycan chains of the ECM possess a highly negative charge [85] that even in small quantities can influence the transport of charged particles [86]. Therefore, for charged particles, electrostatic interactions with the ECM components could be a limiting force [82].

Ex vivo studies by Lieleg et al. [23] on ECM isolated from mice sarcoma have shown that the presence of surface charge on polystyrene particles (irrespective of positive or negative) significantly suppresses their diffusion. They further reveal that by tuning the strength of the interactions between particles and the ECM via masking surface charges, the local mobility of otherwise trapped particles could be restored. They masked the surface charges of negatively charged particles with the addition of salt (1 MKCl) or positively charged particles with polyanionic proteins (0.5 mM Heparin), which hints at the role of ionic interactions between particles and ECM components [87]. Braunger et al. [88] used an in vitro flow-based device containing ECM gel to investigate particle interactions with ECM. They used two types of polymer-based particles—nearly neutral poly(ethylene glycol) (PEG: −4±2 mV) and negatively charged poly(methacrylic acid) (PMA: −38±5 mV)—and attributed the negative surface charge to PMA to induce attachment to the positively charged patches of ECM. Such findings, though not conclusive in terms of predicting particle flow in relation to surface charges, still highlight the important role of filtering via electrostatic interaction by ECM components for drug delivery.

It is worthy to note that existing studies on particle mobility in isolated ECM or mimicked materials (hydrogels) are restricted to investigations of diffusion-based transport only. In the current literature, the transport of charged particles in CNS tissue via infusion-based delivery remains out of focus. To the best of our knowledge, there are no systematic reports accounting for the influence of pressure during charged particle flow under a positive pressure gradient in brain CNS tissue. However, considering molecular processes, pressure can be an important factor. Hydrostatic pressure is well known as a thermodynamics and kinetic variable for biomolecular systems such as lipid bilayers [89]. Moreover, it is known that an altered expression of ECM molecules ultimately affects biochemical processes [81]. This suggests that an applied pressure may affect the electrostatics involved in the ECM filtering mechanism and consequently particle flow behaviour.

The interactions between particles and tissue components are not limited to only ECM, and it is important to consider neurons and glial cells as well. The importance of surface chemistry in particles’ interactions with cells has been well documented [90]. The ability of cells to interact via molecular interactions with surrounding objects based on their specific physiochemical characteristics, e.g., NPs [91,92], is important to consider. A review on molecular-level interactions between engineered materials and cells can be found at [93]. Physiochemical characteristics, e.g., integrins (cell anchoring molecules) binding to ECM or other cells, are well studied and are known to affect cellular processes such as growth and development [94,95]. Electrostatics play an important role, e.g., higher cellular uptakes of NPs have been correlated to their positive surface charges, which facilitate interaction with negatively charged cell membranes [96,97,98]. Such interactions can be mathematically modelled. For example, the framework developed by [99] that considers aspects of particle-cell, particle-microenvironment, and particle-particle interactions.

This understanding also becomes relevant when considering neurons and glial cells in localised drug delivery in CNS. In the case of infusion-based drug delivery, besides the biomechanical resistance, neurons and their elongated axons and glial cells can also influence flow and distribution behaviour through various interactions with drug particles. The adhesion, cellular uptake and aggregation of particles can reduce the concentration required at the target site for the maximum efficacy of the drug. However, it is not known how the infusion pressure would affect this all. This emphasises how much less we know when it comes to associated molecular processes in infusion-based targeted drug delivery and its link to the other aspects of the process, at least when the focus is to design the entire workflow to enable the delivery and maximise the drug reach and uptake. Thus, it will be critical to introduce particle interactions with CNS components in future infusion-based drug delivery research and pursue an improved understanding of the mechanisms controlling drug flow and uptake from both experimental and modelling perspectives. Only by shedding light on such molecular processes will it be possible to develop realistic models for infusion-based drug flow in CNS. Such models can then be incorporated to optimise CED-like systems to maximise the chances of success of infusion-based neurosurgical procedures.

## 4. Simulations and Existing Models for Infusion-Based Drug Delivery

### 4.1. Mathematical Models

Mathematical modelling has been playing an important role in the development of CED. There have been many theoretical models established to understand how fluid flows in the brain parenchyma and how brain tissues deform accordingly. Depending on the research objectives, these models mainly fall into three categories, namely single-phase models, biphasic models and multiphasic models.

#### 4.1.1. Single-Phase Model

In studies where the efforts to theoretically improve CED were primarily focused on predicting the flow field in the brain, Darcy’s law and computational fluid dynamics (CFD) are the major theories and methods to build the single-phase models. An example of pioneering work is [100], where the authors used a magnetic resonance imaging (MRI) technique to reconstruct a 2D brain geometry; through solving convection–diffusion equations, they precited the transport of interleukin-2 in the brain parenchyma after perfusion. They argued that the brain microstructure and transport properties of drug molecules have a great effect on drug transportation and distribution in the brain.

Years later, a more advanced image-based 3D brain model was developed, which allowed for considering and comparing different areas of the brain [24]. The authors also stressed the importance of establishing anisotropic and microstructurally heterogeneous models since they found significant differences of the drug distribution in white matter and grey matter, along and perpendicular to nerve fibres. Linninger’s group [40,101,102] and Kim’s group [103,104,105] then conducted a series of studies to explore the methods of obtaining anisotropic and heterogeneous models of brain by using diffusion tensor imaging (DTI). They also considered chemical kinetics in the new models, which further improved the accuracy of drug flow prediction in brain tissue and provided more reliable suggestions on the design of catheters for CED.

Hydraulic permeability (κ) is a vital parameter in these models as Darcy’s law [106] serves as the most fundamental theory. κ of brain tissues is usually measured by experimental tests, but the tested values are distributed in a wide range, as discussed in Section 3.1. As an alternative, Vidotto [36] established a CFD approach to characterised κs of corpus callosum, superior longitudinal fascicle and uncinate/inferior occipitofrontal fascicle, three different regions of white matter. Based on this method, with the aid of an advanced diffusion imaging technique, namely the Neurite Orientation Dispersion and Density Imaging (NODDI), the authors further established an anisotropic permeability tensor in real brain geometry to predict drug distribution after CED [107].

Single-phase models have been very sophisticated after nearly 20 years of development, especially with the aid of advanced imaging techniques in building realistic geometries. However, they have an obvious limitation as they neglect the tissue deformation during the infusion process. In reality, the neuron and glial cells together with ECM, which constitute brain tissues, are extremely soft in nature (E is lower than 20 kPa for cells [108,109] and a few hundred Pa for ECM [74]) which means that brain tissues are very easily deformed under the hydraulic pressure, as discussed in Section 3.1. Therefore, to obtain more precise predictions of the flow field in brain tissue, it is important to consider the biphasic nature of brain tissue.

#### 4.1.2. Biphasic Model: Fluid Flow and Solid Response

**Figure 3:**
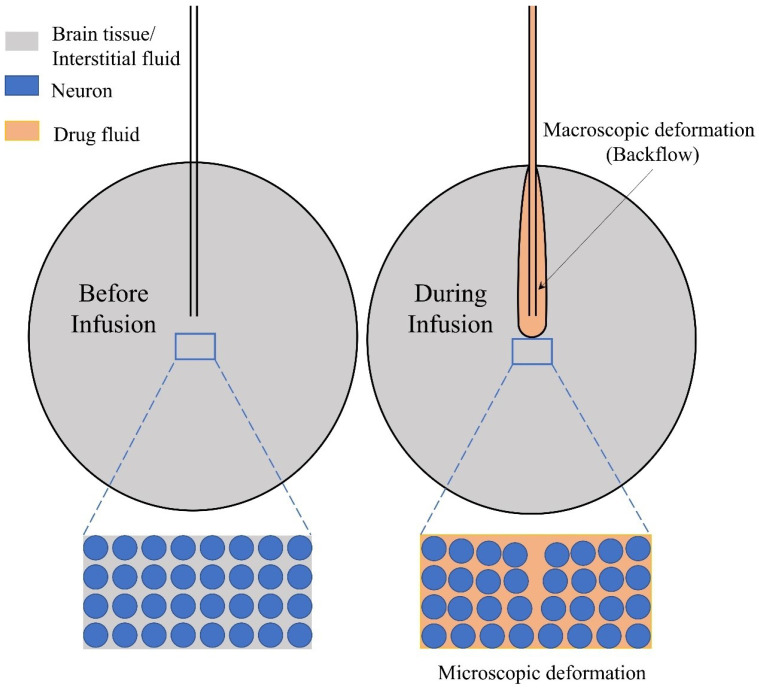
Schematic representation of macro- and microscale deformation of brain parenchyma during infusion.

The tissue deformation is classified into two categories: macroscopic and microscopic deformation (as illustrated in Figure 3). While macroscopic deformation is mainly represented by the phenomenon of backflow, microscopic deformation is to consider the deformation of microstructure that can change the local flow field. In 2002, Chen et al. [110] established the poroelastic model of brain tissue for the purpose of providing more accurate prediction of fluid flow in brain tissue via CED. Apart from being able to visualise the flow field in the brain tissue, the microstructural deformation driven by the localised hydraulic pressure could also be considered. Experiments with brain-like agarose gel were conducted to validate the model. As this was the initial stage to build the poroelasticity model for brain tissues, some hypotheses were applied, such as the linear elasticity, isotropy and homogeneity.

To overcome the limitation of linear elasticity hypothesis, ref. [34] adopted an exponential form of deformation-dependent hydraulic permeability which was obtained from agarose gels and biological soft tissues such as cartilage. Apart from using empirical formulas, introducing nonlinear material properties to the model is also an effective approach and has been thoroughly investigated, especially in Smith’s group. They firstly described the brain tissue by an Ogden-type hyperelastic compressible function so that the nonlinear correlation between the local hydraulic permeability and the local hydraulic pressure could be modelled [111]. Then, the geometrical nonlinearity was also added to the model so that the hydraulic permeability depends on both the matrix strain and nonlinear boundary conditions at the infusion cavity [112]. By comparing the prediction difference between the single-phase model and the biphasic model, they highlighted the significance of considering the finite deformation of the matrix in predicting drug distribution patterns and concentration distribution. Their studies also indicated the importance of experimental determination of material nonlinearity of brain tissue over a range of strains [112,113].

Another direction to improve the poroelasticity models is to consider the anisotropy and heterogeneity of brain tissues. The pioneering work was conducted in 2012 by [38], where the authors utilised DTI to obtain patient-specific brain structure and parameters and implemented the data in the biphasic model. The difference between white matter and grey matter was also characterised by fractional anisotropy. However, the material nonlinearity was not considered in this model.

Backflow is one of the main drawbacks of CED as it would significantly decrease the drug volume and should be avoided [114,115,116]. To model this phenomenon, a fluid–solid interaction method should be an ideal option as this involves overall deformation of the tissue driven by the flow, and there is a clear boundary between the solid phase and the fluid phase where the fluid–solid interface develops. However, fluid–solid interaction has not been applied due to numerical challenges introduced by the unknown time-varying hydraulic pressure between the tissue and the catheter [116]. Some alternative methods were then proposed. One method was to use two layers of solid elements to represent the forward flow and backflow, respectively [117]. Benefitting from the deformability of the two layers of solid elements, the deformation of fluid–solid boundaries was able to be described. This method was also adopted and extended to 3D in [116]. The most important factors that determine the accuracy of this treatment are the properties of these two layers of elements, which require the elements to be able to possess fluidic behaviours. Another method is to load the hydraulic pressure directly on the solid domain and monitor the solid deformation [114]. Therefore, determining how to obtain the hydraulic pressure is one of the most important steps.

#### 4.1.3. Multiphasics Model

Other fluids or components, such as the CSF and IF, may also affect the flow field of the drug inside brain tissues, thus constituting the other phases of the mathematical model. In Stine’s review [118], the relationship between CED and IF dynamics was thoroughly analysed. By integrating pulsatile CSF motion and intracranial pressure into the biphasic model, Linninger’s group established a prototype of a more systematic model [119,120]. Although the original purpose of developing this model was to study hydrocephalus and no external fluid was considered in this model, it could be adapted for CED simulation. Later, transvascular fluid exchange was added to a nonlinear biphasic model to present a more sophisticated system for analyses of flow infusion in the brain [121]. To be more realistic, IF, blood plasma [122,123] and tumour leakiness [124] were also taken into account. In Zhan’s model [125,126,127,128], the whole journey of drug transport in the brain, including drug release, extracellular exchange and intercellular reaction, was considered together with the drug fluid, IF and blood. By modelling the most realistic situation, this model has great potential to provide holistic pre-operative suggestions.

### 4.2. Mathematical Models for Particle Distribution in CNS

The distribution of drug particles (e.g., NPs) in brain parenchyma via diffusion is also a major factor that determines the efficiency of drug delivery [57]. Thus, understanding how NPs diffuse in the extracellular space (ECS) of the brain is vital for not only the development of drugs for brain diseases but also the pre-operative planning of CED treatment. The diffusivity of NPs in brain tissues is, in fact, the macroscopic and statistical expression of particle behaviours at the micro scale, which is governed by biophysical and biochemical particle–tissue components (TCs) interactions. Therefore, in theory, the diffusivity of NPs in CNS can be mathematically predicted if the particle–particle and particle–TCs interactions can be properly defined. However, progresses and applications in this direction are rare. The major factors are lack of brain microstructure with high resolution and accuracy of existing data. The accurate description of particle–TC interactions depends highly on the spatial relations between the particle and the TCs. Despite this limitation, some mathematical models based on idealised geometries have been established. In Nicholson’s group, which has been devoted to unveiling the mystery of brain ECS [16,129,130,131,132,133], three different types of idealised ECS geometries were constructed, within which 3D Monte Carlo (MC) simulations were performed and the diffusivity of the particles was calculated statistically [134]. Through this study, the geometrical hindrance of ECS on particle diffusion was revealed. Years later, the 3D simulation scheme was reduced to be a quasi-1D method to avoid heavy consumption of computational resources, which might promote wider applications of this method [135]. Some other groups also used the MC method to model particle diffusion in simplified brain geometries. For example, Fieremans et al. [136] combined a fibrous geometry of different packing patterns and packing densities with MC simulation to show that fibre phantoms are able to quantitatively validate the diffusion image on clinical MRI scanners. Nilsson et al. [137] reported how axonal undulation manipulates the diffusion of water molecules. Although the aims of these two works were to improve the accuracy of MR imaging, the mathematical methods and the simplified geometric models were worth considering in the prediction of drug diffusion in CNS.

Another direction of mathematical modelling work is using a single particle tracking algorithm, which can calculate the real trajectory of every single particle and then derive the diffusivity of the drug particles by Equation (1)

(1) $$D=<R^{2}>/6t,<R^{2}>=\sum_{i=1}^{n}\left(dx_{i}^{2}+dy_{i}^{2}+dz_{i}^{2}\right)$$

where $<R^{2}>$ is the average of mean square displacement (MSD) of all the particles. *dx*, *dy* and *dz* are the displacement of an NP in the *x*, *y* and *z* directions, respectively. *n* is the number of NPs in the system. *t* is the diffusion time.

Su et al. [138,139] have carried out some pioneering studies on exploring the ability of the mathematical particle tracking method to estimate the diffusivity of NPs in tissue. The model was able to consider particle–cell interactions and the consequent results, such as particle deposition. However, the object was cancer tissue, and no brain properties were considered. If brain microstructure can be properly constructed, this method may also possess great potential to predict NP diffusion in CNS. The framework developed by [99] contains stochastic reconstructing geometry of brain white matter and a mathematical particle tracing model. Based on this framework, they were able to investigate the relationship between surface charge, size and the effective diffusion coefficient of NPs in brain white matter.

### 4.3. Modelling Molecular Interactions

An important aspect of NP transport mechanisms in the brain is to investigate their interactions with TCs at the molecular scale. Adopting a suitable modelling approach can indeed provide valuable information on the effect of interactions at the molecular level. Approaches based on CFD and molecular dynamics (MD) simulations are not best suited for this task because the former deals with higher length and time scales, which are not capable of describing molecular interactions, and the latter can only consider a limited number of atoms and very short time scales (order of nm), which are not sufficient to describe the interaction between tissue components and complex drug molecules [140,141]. The coarse-grained molecular modelling approach via representing a cluster of atoms as a single bead, e.g., using dissipative particle dynamics (DPD) [142], allows for larger timesteps to be taken and is therefore appropriate for tackling such meso-scale problems. This can allow for investigation into how certain parameters of NPs and various physical characteristics of the tissue components affect molecular interactions, so as to allow a range of drug particles and environments to be modelled accurately. In the DPD approach, NPs can be simulated in the form of micelles and the axon’s surface as bilayer membranes to investigate adherence between them. Considering the role of the drug concentration–time profile in its effectiveness, it is desirable for the drug to attach to the targeted site of tissue only. This can be investigated in DPD by adopting a single bilayer comprised of two portions—attractive and repulsive—and determine factors that could possibly enhance or detract away from these localised interactions, such as bilayer geometry or micelle size in a confined space. These insights could thus help in the formulation of larger-scale descriptors and models. The DPD does have the ability to produce these more complex structures [143], but it is common to employ a bottom-up methodology in obtaining interaction parameters in order to match properties to a particular surfactant or drug molecule. If a standardised drug carrier were to be adopted for the CED process, the time investment to obtain such parameters from either experimental data or MD simulations would be justified by reducing the time spent matching parameters as with the top-down approach and extremely time-consuming trial-and-error adjustments and re-formulation of drugs.

### 4.4. Geometric Models

Along with the development of mathematical models is the updating of geometric models. The development and applications of geometrical models can be categorised into two types, namely idealised models and image-based realistic models.

#### 4.4.1. Idealised Models

On the initial stage of model development, idealised geometric models are usually used to test the mathematical model. For example, based on brain phantom gels, which can be treated as an isotropic and homogeneous medium, Chen et al. [110] established the poroelastic biphasic model in 2002. The upgrading work to add material and geometric nonlinearity was also based on an idealised spherical model [34,111,112,113,121,139]. An idealised model is suitable to conduct parametric studies to investigate the effects of different material and infusion parameters on drug distribution because geometrical properties are not the main focus. Therefore, in Zhan’s work that studied the effect of tissue permeability and drug diffusion anisotropy on CED [57], and in Su’s work that investigated how infusion rate, needle diameter and tissue properties affected the development of backflow, spherical models were also adopted [139]. It is also worth mentioning that, so far, almost all the geometrical models used to simulate particle diffusion were idealised models [135,137,138,139]. Some of the major reasons that might have influenced this are: (i) the lack of anatomical images with high resolution and fidelity to reconstruct the realist microstructure of brain tissue; (ii) complexity of the realistic microstructure and microenvironment will lay an extremely heavy burden on computational resources to solve the mathematical equations.

#### 4.4.2. Realistic Models Using MRI and DTI as Input

Owing to the role of anisotropy and heterogeneity of brain tissues, the employment of more realistic geometric models is necessary if an accurate prediction is needed at the relevant length scales [24,57]. Therefore, the past 20 years have seen vast developments and applications of anatomical MRI and DTI techniques in the simulation works. The earliest work could date back to 1997 when Kalyanasundaram [100] used MRI to build a 2D realistic brain geometry. Soon after, the 3D geometry of the brain was also successfully reconstructed with the aid the anatomical MRI [24]. When the importance of anisotropy was realised, the DTI technique also began to be widely applied to characterise the direction of nerve cells, especially the axons in white matter and anisotropy of brain tissue [40]. From then on, the utilisation of anatomical MRI and DTI techniques to reconstruct brain tissue became the routine method in the application of mathematical modelling [38,103,104,105,123,124,126,127].

The ultimate goal of developing mathematical models is to provide suggestions on pre-operative planning. However, due to individual variation, one geometric model cannot be used for another patient. Therefore, obtaining patient-specific models is also a hot topic and has been widely explored [38,144,145,146,147].

### 4.5. Limitations and Perspectives

Although mathematical and geometric models for CED have been improving consistently in the past 20 years, they still need perfected to meet clinical requirements.

#### 4.5.1. On Mathematical Models

The theory of poroelasticity in geoscience is a great inspiration and powerful tool in the study of mechanical behaviours of saturated porous tissues. However, being different from soil and rocks, the microstructure in biological tissues is far more complex [16,130,131]. While assuming localised homogeneity and isotropy agrees well with geology experiments [148], the same hypothesis may not applicable for brain tissues [18]. To achieve more accurate outcomes, it is worth moving to a microscopic scale in order to analyse the interaction between the hydraulic pressure and microstructures, which also raise the need for a reconstruction method with higher resolution imaging techniques [19,58]. It is also worth mentioning the mathematical models for backflow simulation here. As of now, backflow cannot be modelled phenomenologically by fluid–solid interaction methods due to the continuously evolving fluid–solid interfaces. Alternative methods may be able to provide reliable predictions, but rely heavily on proper determinations of the elemental properties in the two layers of solid elements [116,117] and the pre-stress on the solid phase [114]. Therefore, building a more intuitive model to simulate backflow needs further consideration. For the particle tracking method, there is another obstacle which needs to be highlighted. The mainstream particle tracking algorithms and software packages treat particles as points without considering the real surface [149]. However, the width of ECS, or the gap between axons, is about 100 nm [150], which is comparable to the size of NPs. Under such conditions, simplifying the particles to be points would lead to large errors because the relatively high-frequency collision between the particle surface and cells membrane or ECM components is not considered by these simplified models. Therefore, particle tracking algorithms still need to be improved before they can provide quantitative predictive capabilities in the simulation of particle diffusion in CNS.

#### 4.5.2. On Model Compatibility

As analysed above, single-phase models show the best compatibility with advanced imaging-based realistic geometries, but not being able to consider the effect of tissue deformation on the drug distribution is a major deficiency. Biphasic models are indeed able to consider the coupling effect of solid phase and fluid phase, but they have not been widely integrated with the realistic geometric model. The reason may be due to numerical challenges and computational burdens. More difficulties might be encountered when embedding multiphase mathematical models into patient-specific brain structures, though this will provide the most reliable predictions. In parallel to exploring the most accurate approach, how to balance the benefits from advanced mathematical and geometric models may also need to be thought out at the current stage.

## 5. Robotics Solutions

Neurosurgery is a field that can greatly benefit from robotic solutions [151,152,153], not least because of the rich history of neurosurgical innovation in stereotaxy, the constrained anatomical environment, the microsurgical nature of procedures, the highly technical nature of the field, the need for growth in minimally invasive surgery and a culture that adopts and embraces new technology [154]. However, general system solutions are rare, likely due to the inherently complex nature of procedures. Early robotic neurosurgical platforms served as computer-assisted stereotactic guidance systems. Indeed, the first medical robotic demonstration in 1985 used a PUMA 560 Industrial robot to guide a brain biopsy needle to a target along a straight trajectory [155]. In 1991, a later version of the system allowed the successful resection of deep benign astrocytomas in six children without morbidity or mortality [156]. Currently, the Renishaw neuromate^®^ stereotactic robot is a commercially available five Degrees-of-Freedom (DoF) serial manipulator system [157] suitable for a broad range of procedures (e.g., deep brain stimulation), including precise deployment of CED infusion needles [158]. Commercial robotic platforms for neurosurgical procedures are an alternative solution to a conventional stereotactic frame or neuronavigation system, with benefit for repetitive tasks (e.g., insertion of multiple electrodes), but still, they provide little improvement on the level of dexterity during needle insertion. Straight needles limit clinical options toward reaching the Region Of Interest (ROI) safely, performing vessel avoidance and following anatomical structures.

Robotic steerable needles [159,160] have the advantage of addressing limitations of straight needles, with the further advantage of allowing the operating surgeon to reach the ROI with a specific orientation and while avoiding obstacles (sulci, vessels, delicate areas) along the way. On the other hand, these mechatronic systems require additional instruments to precisely navigate into the brain and reach the planned target with sufficient accuracy (compared to experienced physicians performing procedures using rigid needles [161,162] as described in [163]). There are three main aspects to consider for an accurate insertion: (i) planning a safe and feasible path to reach an ROI, (ii) tracking the position of the needle’s tip during the insertion, (iii) tracking changes and deformations happening in the brain during surgery.

### 5.1. Planning

There are two main types of planners required to successfully organise and perform steerable needle insertion into the brain: preoperative and intra-operative.

The preoperative planner should consider different types of information (target pose, clinical obstacles to avoid, kinematics constraints of the needle) and give the surgeon a choice of feasible paths that satisfy all the constraints. The many possible solutions can be evaluated and ranked on the basis of specific optimal criteria [163,164]: minimisation of the surgical path length and maximisation of the distance from anatomical obstacles are mostly used in current practice. In the literature, there are many different approaches to preoperative planning of steerable needles. Besides more recent attempts exploiting evolutionary strategies [165] or machine learning techniques [166], the majority of the state-of-the-art planners are graph-based methods, such as A* [167,168], or sampling-based methods, such as Rapidly exploring Random Trees (RRT) [169,170,171,172] and Adaptive Fractal Trees (AFT) [173,174]. In [175], the authors presented a new searched-based planner based on [176,177] for steerable needles that guarantees completeness under some clinically reasonable assumptions. This is important because having the formal proof that if a plan exists, it will be found and if it does not exist, the user will be notified may prove to be an important aspect for medical certification.

The intra-operative planner must take into account the same criteria that were applicable during the preoperative phase, with the additional constraints of the computation time limit and tissue deformation affecting the needle insertion process, which would lead to a change of the safety distance from obstacles if not accounted for. The time bounds for each computation can be tight and depend on the insertion velocity of the needle and the sensory computation time needed to localise the needle. On the other hand, the search space is usually smaller than in the preoperative case, because of the nonholonimic kinematic limitations of the needle and the overarching aim to maintain high “path similarity” to the original, preoperative plan. Path similarity is a measure of the deviation between the path evaluated and initially chosen by the surgeon and the new path taken by the needle during the insertion. The approaches are like those for preoperative planners, but with specific optimisation to limit computational time. In [163], a modified RRT algorithm for rapid replanning is presented, and it is integrated in the robotic system as control algorithm. The same approach is followed in [178], with a modified version of the bubble-bending algorithm [179,180] and a 3D extension of the Convex Elastic Smoothing presented in [181].

### 5.2. Needle Tracking

Conventional neuro-navigation systems [182,183,184] use stereotactic frames or optical trackers to identify the position of the tip of the needle knowing the entry point in the skull and the angle of insertion. This is not enough for steerable needles. In many technological solutions, such as Programmable Bevel-Tip Steerable Needles (PBNs), the kinematics of the needle and the dynamic interaction with the tissue of the brain make it difficult to rely only on mathematical models for tracking. In these cases, some type of sensing is required to provide accurate feedback to the robotic control system and the visualisation to the user.

Intra-operative MRIs are used for CED with rigid needles [185] and other applications with steerable catheters [186,187]. MRI is usually preferred over computed tomography (CT) because it does not expose the patient to ionising radiation. On the other hand, the robotic system should be designed to be MRI-compatible, with some solutions like PBNs [188] more suitable than others. There are also CT-based solutions when ionising radiations are not a concern [189]. Both approaches tend to be slow [190] and require a complex setup.

Among others, the most promising alternative sensing system available for needle tracking is Fiber Bragg Grating (FBG)-based optical fibres, or FBGs for short [191]. FBGs can be easily embedded into lumens to provide information such as the full shape reconstruction of the needle, hence providing a wealth of geometrical information, including the pose of the tip [192,193,194].

Another alternative intra-operative tracking modality is ultrasound (US), which has been shown to be effective for neurosurgical procedures such as biopsies [195]. Ultrasound has many advantages over other imaging modalities: it is less expensive, easy to setup and use and has a very good temporal resolution, and it is already in use for guiding needle insertion [196]. However, to be used for neurological applications (including CED), it is invasive since it requires contact with the dura, if wide, unimpeded and appropriately resolved volume data are required. There are other reasons to use US in this type of surgery, as explained below. In these cases, US can be used also for tracking, especially together with other sensors, including FBGs, to increase accuracy [197].

### 5.3. Brain Deformation Sensing

During brain surgery, significant deformations can occur due to brain shift or tool–tissue interactions. This means that preoperative plans are not reliable intra-operatively because the patient-to-image registration becomes invalid [198]. In previous subsections, intra-operative planners were presented to adjust tool paths during the insertion process. To compute a new safe path, complete knowledge of the deformation affecting the target and clinical obstacles is desirable. MRI and CT have enough spatial information for the planner, but they are slow and often cumbersome to use intra-operatively, as previously stated. Trans-cutaneous US (through an additional burr hole in the skull) would offer an appropriate update time and portability, but it lacks resolution. One solution is to use fast 2D/3D US information to deform preoperative high-resolution images acquired with another higher-fidelity imaging modality.

In the literature, there are many methods for MRI/US registration, including linear correlation of linear combination LC2 [199], block-matching approach [200] and self-similarity descriptors [201]. These approaches must be able to manage 2D/3D US images in real-time. In [202], a modern open-source graphic processing unit (GPU) accelerated ultrasound processing platform is presented. Another approach is to develop models that can accurately predict the deformation of the brain during neurosurgical procedures. For example, Forte et al. [203] developed a numerical model for the prediction of brain shift during surgical procedures and employed a phantom made of a composite hydrogel [204,205] to validate their findings. Such studies, combined with the use of advanced experimental investigations [41,61,206], highlight the importance of modelling using robust and fully validated constitutive laws for the simulations of brain deformation [42,47,207]. The use of reduced-order models derived from more complex descriptions of the tissue deformation has also been pursued and holds promise in the context of guiding intra-operative adjustment and helping clinicians during surgical procedures [208,209].

### 5.4. Software

Complex tasks such as surgery planning, initialisation and control of a robotic system require a reliable and solid software suite. Currently, there are no commercial systems for CED that are based on steerable needles, so the only actual implementations are prototypes developed for research purposes.

In [9], the current state of open source for robotically steerable needles is presented, listing software tools that can be used for the planning of the operation and any computation required, such as 3D Slicer [210] or the Medical Imaging Interaction Toolkit (MITK) [211]. There are also open protocols that can be used to transfer medical images, transformations and parameters from different computational units, such as OpenIGTLink [212]. Finally, there are more traditional robotic frameworks for simulation and control such as the Simulation Open Framework Architecture (SOFA) and Kitware’s Interactive Medical Simulation Toolkit [213,214]. They are all valuable instruments to create ad-hoc solutions starting from a solid and tested base. Yet, there are some aspects of a robotic surgical platform that are not completely addressed by those tools.

Another critical aspect of a medical system is the user interface (UI). In fact, the UI should be usable without ambiguity by clinicians, and this requires a careful design of the graphical elements of the interface and a deep understanding of the type of information that is important to the user. Companies providing neuro-navigation systems [182,183,184] usually have well-designed and easy-to-use UIs. In some research CED projects [215], a standard commercial system is used for clinical assessment or interventional planning, as is importing or exporting files between other research software components. In [160], a proper communication protocol was developed in collaboration with Renishaw plc (a partner of the project) to enable data exchange between a custom version of the neuroinspire^TM^ suite and the computational back-end running the steerable catheter and sensors computation. The UI has been designed with the same User Experience (UX) design principles of the standard navigation interface that is already familiar to radiologists and surgeons. The need to drive a steerable catheter in a three-dimensional space, while providing information about anatomical structures, eloquent areas and navigation information, on the other hand, is not part of the standard user experience of conventional neuro-navigation software. In [216], a pilot study to evaluate, both qualitatively and quantitatively, a novel human–machine visual interface for the steering of a PBN was carried out. Annotated axial, coronal and sagittal planes from available medical images are displayed together with a manned/unmanned aerial vehicle-inspired [217,218] 3D first-person viewpoint when navigating the needle. Visual cues are used to convey additional information, such as the deviation from the planned trajectory and prediction about the motion of the needle. The study provided good results, in terms of qualitative evaluation of the interface and quantitative evaluation of the performance of the subjects in following a 3D path.

## 6. Clinical Implications

Imaging plays a key role in planning and performing convective drug delivery in the brain. Conventional and advanced imaging techniques provide essential morphological, physiological and metabolic details of the targeted pathological tissue and of its adjacent neural and vascular structures [219,220]. A review of the most relevant preclinical and clinical studies using CED for GBM identifies optimal catheter positioning and detailed analysis of drug distribution as major challenges associated with CED [221]. As for cannula placement, the PRECISE trial is the largest study to date using CED for the delivery of a genetically engineered therapeutic agent for the treatment of patients with recurrent GBM [215]. Despite intensive training, in this trial a majority of catheters were not positioned correctly, highlighting the complexity of this technique. Detailed analysis of catheter positioning score, overall placement score and imaging change score did not reveal any correlation of these parameters with clinical outcome. As such, further trials using CED as a delivery method should incorporate advanced imaging, even using real-time intra-operative imaging, to confirm adequate catheter positioning and accurate drug distribution, ensuring that a targeted brain area is adequately covered [222]. Imaging tracking also enhances safety by confirming that drug delivery is limited to the targeted site. Furthermore, data derived from infusions can be used to directly inform predictive drug delivery models, which could optimize catheter placement in the future by proving convective delivery properties in vivo. In this regard, the possibility to virtually dissect white matter fibers by the DTI-based tractography technique allows to consistently reconstruct the course of eloquent tracts in the brain [223] and depict pathological involvement [224]. While tractography is widely employed in the presurgical evaluation of brain tumor resections [225], and it has recently proven its relevance in the planning of tomotherapy radiation treatment [226] and Deep Brain Stimulation [227], it is not currently fully integrated into the pre-operative planning and intra-operative targeting system of CED procedures neither in clinical studies on human subjects nor in most preclinical studies on animals conducted in swine [228], monkey [229], ovine [230] and rat models [231]. The few exceptions are represented by works, where a precise brain structure was targeted according to tractography reconstructions [232], or the relationship between the tissue properties and the infusate was investigated [105,146,233]. A meticulous definition of the brain microstructure in the exact location of infusion would be impactful to better understand and possibly predict the actual geometry of the spatial distribution of a drug in a given patient [38,145,234,235], and also to depict the pathological rearrangement determined by the lesion and previous treatments, such as surgery or radiation therapy. Such pre-surgical infusion planning would be essential to apply CED technological platforms to patient-specific clinical use cases, given the relevant structural and temporal heterogeneity of brain tumors, that may also disclose inter-subject variability according to a patient’s therapeutic history. In addition, advanced imaging techniques would also be essential, along with the clinical status, to assess the response to therapeutic infusion, as current disease parameters have imaging as an essential parameter of response. Ultimately, the outcome of CED procedures is influenced by several factors, such as catheter design and placement; tumor site, size and microstructure; infusion parameters (rate, frequency, volume); drug chemical structure and concentration; and brain morphology. To increase the likelihood of clinical impact, these parameters should all be regarded as essential and then tailored to the subject, according to the clinical history and actual pathological condition. Finally, it would be essential to take advantage of both mathematical modelling and diagnostic accuracy in describing tumor and unaffected brain tissue microstructure to better understand how brain flow pathways affect both tumor preferential tissue invasion and drug distribution [118].

## 7. Conclusions and Future Perspectives

Brain diseases cost the European Economy approximately 35% [236,237,238] of the overall disease burden. Of all brain diseases, brain tumours are comparatively rare, but very costly per case [239]. GBM, for instance, affects 6 out of every 100,000 people. The location of the disease and its biology pose major challenges for effective treatment [240], which has resulted in a lack of improvement in mortality rates during the last 20 years. Even in cases in which surgery is possible, the excision has to be relatively conservative in order to prevent potential damage to the surrounding healthy brain. Due to this requirement, the tumour cannot always be fully eliminated. Chemotherapy is also of limited treatment value for GBM because of the BBB, which impairs drug delivery [241,242]. The US Brain Tumor Foundation estimates that the cost of treating a GBM is between $450,000 and $700,000 in a lifetime. However, the investment in brain-cancer-related research in Europe remains very low in absolute value (approximately ten times lower than for affective disorders) and in comparison to the United States. Furthermore, over two thirds of European brain cancer research funding is privately invested by pharmaceutical companies in the quest for new drugs [239]. As an illustrative number, the estimated average cost of developing a new pharmacological agent to improve cancer patients’ quality of life is in the order of 1 billion euros. A similar investment is needed in the modelling of disease processes and drug delivery mechanisms and on advanced technologies for the accurate administration of targeted drugs through minimally invasive approaches.

This review has covered the fundamental aspects of research that have been performed in the field of drug delivery in brain tissue, with particular focus on techniques that require drugs to infuse and diffuse into the tissue. We have highlighted the limitations that must be addressed in order for the research community to be able to tackle the outstanding important issues which have hindered progress in this field. A synergistic approach is needed, whereby the molecular level mechanisms, which control the chemical processes that regulate drug uptake as well as biomechanical aspects of drug flow and reach within the tissue, must be studied and linked to larger-scale models and experiments in order to better capture drug delivery mechanisms at the macro scale using a bottom-up approach. This will enable to better predict the outcome of, e.g., CED procedures, especially when these are assisted by the development of state-of-the-art minimally invasive devices for robotic surgery and accompanying software, hence providing clinicians with important tools to plan and optimise such procedures and improve the mortality rate. The technological advancements needed to allow clinicians and brain researchers to achieve a marked improvement in outcomes include, amongst others:

1. Consider tumour growth and expansion according to computational evolution data and, from these data, achieve procedure-optimised therapy plans which account for patient-specific anatomy.
2. Obtain a better understanding of molecular-level processes that govern drug uptake, interaction with the tissue and ultimately drug reach and efficacy.
3. Pair diffusion imaging data with histology, microstructural measurements and high-fidelity diffusion models based on computational fluid dynamics and finite element analysis to improve our understanding of the brain biphasic nature.
4. Develop better bottom-up models and associated experimental techniques that enable to describe drug diffusion and convection across the scales and quantify its uptake considering the complexity of the tissue and its variability.
5. Target the main diffusion tracts along and within the tumour and deploy CED systems that can deliver various molecules in various areas of the tumour.
6. Rely on a single, integrated platform for neuro-oncological treatment and a spectrum of other uses, ranging from degenerative disease management and structural definition to stem cell therapy and localised tissue ablation.
7. Integrate pre-operative imaging with online imaging via save modalities, such as ultrasound, to correct for brain shift and to ensure a more accurate targeting, overcoming most of the limitations of current systems for catheter insertion based on preoperative imaging alone and reducing the cost of those few systems (e.g., MR Interventions’ ClearPoint) which rely on an interventional MRI or CT suite.

It is by solving these outstanding open challenges in molecular level understanding, experimental characterisation, modelling, imaging and medical device development that patient outcomes for a range of untreatable brain diseases will finally take a turn for the better.

## Data Availability

Not applicable.

## Abbreviations

The following abbreviations are used in this manuscript:

AFT	Adaptive Fractal Trees
BBB	Blood–Brain Barrier
CED	Convection-Enhanced Delivery
CFD	Computational Fluid Dynamics
CNS	Central Nervous System
CSF	Cerebrospinal Fluid
CT	Computed Tomography
DPD	Dissipative Particle Dynamics
DoF	Degrees of Freedom
DTI	Diffusion Tensor Imaging
E	Young’s Modulus
ECS	Extracellular Space
ESF	Extracellular Fluid
ECM	Extracellular Matrix
FBG	Fiber Bragg Grating
GBM	Glioblastoma Multiforme
GPU	Graphic Processing Unit
if	Interstitial Fluid
κ	Hydraulic Permeability
MC	Monte Carlo
MD	Molecular Dynamics
MIKT	Medical Imaging Interaction Toolkit
MRI	Magnetic Resonance Imaging
MSD	Mean Square Displacement
NODDI	Neurite Orientation Dispersion and Density Imaging
NPs	Nanoparticles
PBNs	Programmable Bevel-Tip Steerable Needles
PVE	Poro-Viscoelastic
RRT	Rapidly exploring Random Trees
ROI	Region of Interest
SOFA	Simulation Open Framework Architecture
TCs	Tissue Components
US	Ultrasound
UX	User Experience
UI	User Interface

## References

[1] Alphandéry E. (2018). Glioblastoma treatments: An account of recent industrial developments. Front. Pharmacol..

[2] Bush N.A.O., Chang S.M., Berger M.S. (2017). Current and future strategies for treatment of glioma. Neurosurg. Rev..

[3] Olesen J., Gustavsson A., Svensson M., Wittchen H.U., Jönsson B. (2012). The economic cost of brain disorders in Europe. Eur. J. Neurol..

[4] Harder B.G., Blomquist M.R., Wang J., Kim A.J., Woodworth G.F., Winkles J.A., Loftus J.C., Tran N.L. (2018). Developments in Blood-Brain Barrier Penetrance and Drug Repurposing for Improved Treatment of Glioblastoma. Front. Oncol..

[5] Weidle U.H., Niewohner J., Tiefenthaler G. (2015). The blood-brain barrier challenge for the treatment of brain cancer, secondary brain metastases, and neurological diseases. Cancer Genom. Proteom..

[6] Yuan F. (1998). Transvascular drug delivery in solid tumors. Semin. Radiat. Oncol..

[7] Mehta A.M., Sonabend A.M., Bruce J.N. (2017). Convection-Enhanced Delivery. Neurotherapeutics.

[8] Lonser R.R., Sarntinoranont M., Morrison P.F., Oldfield E.H. (2015). Convection-enhanced delivery to the central nervous system. J. Neurosurg..

[9] Audette M.A., Bordas S.P., Blatt J.E. (2020). Robotically Steered Needles: A Survey of Neurosurgical Applications and Technical Innovations. Robot. Surg. Res. Rev..

[10] Terzano M., Dini D., Rodriguez y Baena F., Spagnoli A., Oldfield M. (2020). An adaptive finite element model for steerable needles. Biomech. Model. Mechanobiol..

[11] Vogelbaum M.A. (2007). Convection enhanced delivery for treating brain tumors and selected neurological disorders: Symposium review. J. Neuro-Oncol..

[12] Azevedo F.A., Carvalho L.R., Grinberg L.T., Farfel J.M., Ferretti R.E., Leite R.E., Filho W.J., Lent R., Herculano-Houzel S. (2009). Equal numbers of neuronal and nonneuronal cells make the human brain an isometrically scaled-up primate brain. The J. Comp. Neurol..

[13] Daneman R. (2012). The blood-brain barrier in health and disease. Ann. Neurol..

[14] Daneman R., Prat A. (2015). The Blood–Brain Barrier. Cold Spring Harb. Perspect. Biol..

[15] Hammarlund-Udenaes M., Paalzow L.K., de Lange E.C. (1997). Drug equilibration across the blood-brain barrier–pharmacokinetic considerations based on the microdialysis method. Pharm. Res..

[16] Nicholson C. (2001). Diffusion and related transport mechanisms in brain tissue. Rep. Prog. Phys..

[17] Nicholson C., Phillips J.M. (1981). Ion diffusion modified by tortuosity and volume fraction in the extracellular microenvironment of the rat cerebellum. J. Physiol..

[18] Jamal A., Mongelli M.T., Vidotto M., Madekurozwa M., Bernardini A., Overby D.R., De Momi E., Rodriguez y Baena F., Sherwood J.M., Dini D. (2021). Infusion Mechanisms in Brain White Matter and Their Dependence on Microstructure: An Experimental Study of Hydraulic Permeability. IEEE Trans. Biomed. Eng..

[19] Vidotto M., Bernardini A., Trovatelli M., Momi E.D., Dini D. (2021). On the Microstructural Origin of Brain White Matter Hydraulic Permeability. Proc. Natl. Acad. Sci. USA.

[20] Jain R.K., Stylianopoulos T. (2010). Delivering nanomedicine to solid tumors. Nat. Rev. Clin. Oncol..

[21] Zhou Y., Chen X., Cao J., Gao H. (2020). Overcoming the biological barriers in the tumor microenvironment for improving drug delivery and efficacy. J. Mater. Chem. B.

[22] Bertrand N., Wu J., Xu X., Kamaly N., Farokhzad O.C. (2014). Cancer nanotechnology: The impact of passive and active targeting in the era of modern cancer biology. Adv. Drug Deliv. Rev..

[23] Lieleg O., Baumgärtel R.M., Bausch A.R. (2009). Selective Filtering of Particles by the Extracellular Matrix: An Electrostatic Bandpass. Biophys. J..

[24] Sarntinoranont M., Banerjee R.K., Lonser R.R., Morrison P.F. (2003). A Computational Model of Direct Interstitial Infusion of Macromolecules into the Spinal Cord. Ann. Biomed. Eng..

[25] Prange M.T., Margulies S.S. (2002). Regional, directional, and age-dependent properties of the brain undergoing large deformation. J. Biomech. Eng..

[26] Miller K. (1999). Constitutive model of brain tissue suitable for finite element analysis of surgical procedures. J. Biomech..

[27] Miller K., Chinzei K. (1997). Constitutive modelling of brain tissue: Experiment and theory. J. Biomech..

[28] Mendis K.K., Stalnaker R.L., Advani S.H. (1995). A constitutive relationship for large deformation finite element modeling of brain tissue. J. Biomech. Eng..

[29] Donnelly B.R., Medige J. (1997). Shear properties of human brain tissue. J. Biomech. Eng..

[30] Miller K., Chinzei K. (2002). Mechanical properties of brain tissue in tension. J. Biomech..

[31] Franceschini G., Bigoni D., Regitnig P., Holzapfel G.A. (2006). Brain tissue deforms similarly to filled elastomers and follows consolidation theory. J. Mech. Phys. Solids.

[32] Goriely A., Geers M.G., Holzapfel G.A., Jayamohan J., Jérusalem A., Sivaloganathan S., Squier W., van Dommelen J.A., Waters S., Kuhl E. (2015). Mechanics of the brain: Perspectives, challenges, and opportunities. Biomech. Model. Mechanobiol..

[33] Budday S., Ovaert T.C., Holzapfel G.A., Steinmann P., Kuhl E. (2019). Fifty Shades of Brain: A Review on the Mechanical Testing and Modeling of Brain Tissue. Arch. Comput. Methods Eng..

[34] Chen X., Sarntinoranont M. (2007). Biphasic finite element model of solute transport for direct infusion into nervous tissue. Ann. Biomed. Eng..

[35] Ehlers W., Wagner A. (2015). Multi-component modelling of human brain tissue: A contribution to the constitutive and computational description of deformation, flow and diffusion processes with application to the invasive drug-delivery problem. Comput. Methods Biomech. Biomed. Eng..

[36] Vidotto M., Botnariuc D., De Momi E., Dini D. (2019). A computational fluid dynamics approach to determine white matter permeability. Biomech. Model. Mechanobiol..

[37] Tavner A.C., Roy T.D., Hor K.W., Majimbi M., Joldes G.R., Wittek A., Bunt S., Miller K. (2016). On the appropriateness of modelling brain parenchyma as a biphasic continuum. J. Mech. Behav. Biomed. Mater..

[38] Støverud K.H., Darcis M., Helmig R., Hassanizadeh S.M. (2012). Modeling Concentration Distribution and Deformation During Convection-Enhanced Drug Delivery into Brain Tissue. Transp. Porous Media.

[39] Raghavan R., Brady M. (2011). Predictive models for pressure-driven fluid infusions into brain parenchyma. Phys. Med. Biol..

[40] Linninger A.A., Somayaji M.R., Erickson T., Guo X., Penn R.D. (2008). Computational methods for predicting drug transport in anisotropic and heterogeneous brain tissue. J. Biomech..

[41] Forte A.E., Gentleman S.M., Dini D. (2017). On the characterization of the heterogeneous mechanical response of human brain tissue. Biomech. Model. Mechanobiol..

[42] Budday S., Sommer G., Haybaeck J., Steinmann P., Holzapfel G.A., Kuhl E. (2017). Rheological characterization of human brain tissue. Acta Biomater..

[43] Budday S., Nay R., de Rooij R., Steinmann P., Wyrobek T., Ovaert T.C., Kuhl E. (2015). Mechanical properties of gray and white matter brain tissue by indentation. J. Mech. Behav. Biomed. Mater..

[44] Cheng S., Clarke E.C., Bilston L.E. (2008). Rheological properties of the tissues of the central nervous system: A review. Med. Eng. Phys..

[45] Feng Y., Okamoto R.J., Namani R., Genin G.M., Bayly P.V. (2013). Measurements of mechanical anisotropy in brain tissue and implications for transversely isotropic material models of white matter. J. Mech. Behav. Biomed. Mater..

[46] Feng Y., Lee C.H., Sun L., Ji S., Zhao X. (2017). Characterizing white matter tissue in large strain via asymmetric indentation and inverse finite element modeling. J. Mech. Behav. Biomed. Mater..

[47] Greiner A., Reiter N., Paulsen F., Holzapfel G.A., Steinmann P., Comellas E., Budday S. (2021). Poro-Viscoelastic Effects During Biomechanical Testing of Human Brain Tissue. Front. Mech. Eng..

[48] Vunjak-Novakovic G., Martin I., Obradovic B., Treppo S., Grodzinsky A.J., Langer R., Freed L. (1999). Bioreactor Cultivation Conditions Modulate the Composition and Mechanical Properties of Tissue-Engineered Cartilage. J. Orthop. Res..

[49] Gu W.Y., Yao H. (2003). Effects of hydration and fixed charge density on fluid transport in charged hydrated soft tissues. Ann. Biomed. Eng..

[50] Heneghan P., Riches P.E. (2008). Determination of the strain-dependent hydraulic permeability of the compressed bovine nucleus pulposus. J. Biomech..

[51] Reynaud B., Quinn T.M. (2006). Anisotropic hydraulic permeability in compressed articular cartilage. J. Biomech..

[52] Zhang X.Y., Luck J., Dewhirst M.W., Yuan F. (2000). Interstitial hydraulic conductivity in a fibrosarcoma. Am. J. Physiol.-Heart Circ. Physiol..

[53] Boucher Y., Brekken C., Netti P.A., Baxter L.T., Jain R.K. (1998). Intratumoral infusion of fluid: Estimation of hydraulic conductivity and implications for the delivery of therapeutic agents. Br. J. Cancer.

[54] Milosevic M., Lunt S.J., Leung E., Skliarenko J., Shaw P., Fyles A., Hill R.P. (2008). Interstitial permeability and elasticity in human cervix cancer. Microvasc. Res..

[55] Walhovd K.B., Johansen-Berg H., Káradóttir R.T. (2014). Unraveling the secrets of white matter-Bridging the gap between cellular, animal and human imaging studies. Neuroscience.

[56] Pieri V., Trovatelli M., Cadioli M., Zani D.D., Brizzola S., Ravasio G., Acocella F., Giancamillo M.D., Castellano A. (2019). In vivo Diffusion Tensor Magnetic Resonance Tractography of the Sheep Brain: An Atlas of the Ovine White Matter Fiber Bundles. Front. Vet. Sci..

[57] Zhan W., Rodriguez y Baena F., Dini D. (2019). Effect of tissue permeability and drug diffusion anisotropy on convection-enhanced delivery. Drug Deliv..

[58] Bernardini A., Trovatelli M., Kłosowski M.M., Pederzani M., Zani D.D., Brizzola S., Porter A., Rodriguez y Baena F., Dini D. (2021). Imaging and reconstruction of the cytoarchitecture of axonal fibres: Enabling biomedical engineering studies involving brain microstructure. Res. Sq..

[59] Kroppenstedt S.N., Thomale U.W., Griebenow M., Sakowitz O.W., Schaser K.D., Mayr P.S., Unterberg A.W., Stover J.F. (2003). Effects of early and late intravenous norepinephrine infusion on cerebral perfusion, microcirculation, brain-tissue oxygenation, and edema formation in brain-injured rats. Crit. Care Med..

[60] Vardakis J.C., Chou D., Tully B.J., Hung C.C., Lee T.H., Tsui P.H., Ventikos Y. (2016). Investigating cerebral oedema using poroelasticity. Med. Eng. Phys..

[61] Jamal A., Bernardini A., Dini D. (2022). Microscale characterisation of the time-dependent mechanical behaviour of brain white matter. J. Mech. Behav. Biomed. Mater..

[62] Hosseini-Farid M., Ramzanpour M., McLean J., Ziejewski M., Karami G. (2020). A poro-hyper-viscoelastic rate-dependent constitutive modeling for the analysis of brain tissues. J. Mech. Behav. Biomed. Mater..

[63] Mow V.C., Kuei S.C., Lai W.M., Armstrong C.G. (1980). Biphasic Creep and Stress Relaxation of Articular Cartilage in Compression: Theory and Experiments. J. Biomech. Eng..

[64] Lai W.M., Mow V.C., Roth V. (1981). Effects of Nonlinear Strain-Dependent Permeability and Rate of Compression on the Stress Behavior of Articular Cartilage. J. Biomech. Eng..

[65] Holmes M.H. (1986). Finite Deformation of Soft Tissue: Analysis of a Mixture Model in Uni-Axial Compression. J. Biomech. Eng..

[66] Mak A.F. (1986). The apparent viscoelastic behavior of articular cartilage–the contributions from the intrinsic matrix viscoelasticity and interstitial fluid flows. J. Biomech. Eng..

[67] Mak A.F. (1986). Unconfined compression of hydrated viscoelastic tissues: A biphasic poroviscoelastic analysis. Biorheology.

[68] Wang R., Sarntinoranont M. (2019). Biphasic analysis of rat brain slices under creep indentation shows nonlinear tension-compression behavior. J. Mech. Behav. Biomed. Mater..

[69] Suh J.K., DiSilvestro M.R. (1999). Biphasic Poroviscoelastic Behavior of Hydrated Biological Soft Tissue. J. Appl. Mech..

[70] Cheng S., Bilston L.E. (2007). Unconfined compression of white matter. J. Biomech..

[71] Mehrabian A., Abousleiman Y.N., Mapstone T.B., El-Amm C.A. (2015). Dual-porosity poroviscoelasticity and quantitative hydromechanical characterization of the brain tissue with experimental hydrocephalus data. J. Theor. Biol..

[72] Mehrabian A., Abousleiman Y. (2011). General solutions to poroviscoelastic model of hydrocephalic human brain tissue. J. Theor. Biol..

[73] Ouyang H., Nauman E., Shi R. (2013). Contribution of cytoskeletal elements to the axonal mechanical properties. J. Biol. Eng..

[74] Wells R.G. (2008). The role of matrix stiffness in regulating cell behavior. Hepatology.

[75] Yuan T., Zhan W., Jamal A., Dini D. (2022). On the Microstructurally-Driven Heterogenous Response of Brain White Matter to Drug Infusion Pressure. Biomech. Model. Mechanobiol..

[76] Novak U., Kaye A.H. (2000). Extracellular matrix and the brain: Components and function. J. Clin. Neurosci..

[77] Bandtlow C.E., Zimmermann D.R. (2000). Proteoglycans in the Developing Brain: New Conceptual Insights for Old Proteins. Physiol. Rev..

[78] Nicholson C., Syková E. (1998). Extracellular space structure revealed by diffusion analysis. Trends Neurosci..

[79] Zamecnik J. (2005). The extracellular space and matrix of gliomas. Acta Neuropathol..

[80] Lau L.W., Cua R., Keough M.B., Haylock-Jacobs S., Yong V.W. (2013). Pathophysiology of the brain extracellular matrix: A new target for remyelination. Nat. Rev. Neurosci..

[81] Engin A.B., Nikitovic D., Neagu M., Henrich-Noack P., Docea A.O., Shtilman M.I., Golokhvast K., Tsatsakis A.M. (2017). Mechanistic understanding of nanoparticles’ interactions with extracellular matrix: The cell and immune system. Part. Fibre Toxicol..

[82] Stylianopoulos T., Poh M.Z., Insin N., Bawendi M.G., Fukumura D., Munn L.L., Jain R.K. (2010). Diffusion of Particles in the Extracellular Matrix: The Effect of Repulsive Electrostatic Interactions. Biophys. J..

[83] Li Y., Asadi A., Monroe M.R., Douglas E.P. (2009). pH effects on collagen fibrillogenesis in vitro: Electrostatic interactions and phosphate binding. Mater. Sci. Eng. C.

[84] Mertz E.L., Leikin S. (2004). Interactions of Inorganic Phosphate and Sulfate Anions with Collagen. Biochemistry.

[85] Bhalla G., Deen W.M. (2009). Effects of Charge on Osmotic Reflection Coefficients of Macromolecules in Fibrous Membranes. Biophys. J..

[86] Thorne R.G., Lakkaraju A., Rodriguez-Boulan E., Nicholson C. (2008). In vivo diffusion of lactoferrin in brain extracellular space is regulated by interactions with heparan sulfate. Proc. Natl. Acad. Sci. USA.

[87] Arends F., Baumgärtel R., Lieleg O. (2013). Ion-Specific Effects Modulate the Diffusive Mobility of Colloids in an Extracellular Matrix Gel. Langmuir.

[88] Braunger J.A., Björnmalm M., Isles N.A., Cui J., Henderson T.M.A., O’Connor A.J., Caruso F. (2017). Interactions between circulating nanoengineered polymer particles and extracellular matrix components in vitro. Biomater. Sci..

[89] Winter R., Jeworrek C. (2009). Effect of pressure on membranes. Soft Matter.

[90] Augustine R., Hasan A., Primavera R., Wilson R.J., Thakor A.S., Kevadiya B.D. (2020). Cellular uptake and retention of nanoparticles: Insights on particle properties and interaction with cellular components. Mater. Today Commun..

[91] Mohammad-Beigi H., Hayashi Y., Zeuthen C.M., Eskandari H., Scavenius C., Juul-Madsen K., Vorup-Jensen T., Enghild J.J., Sutherland D.S. (2020). Mapping and identification of soft corona proteins at nanoparticles and their impact on cellular association. Nat. Commun..

[92] Price E., Gesquiere A.J. (2019). Author Correction: An in vitro assay and artificial intelligence approach to determine rate constants of nanomaterial-cell interactions. Sci. Rep..

[93] Jang Y.H., Jin X., Shankar P., Lee J.H., Jo K., Lim K.I. (2019). Molecular-level interactions between engineered materials and cells. Int. J. Mol. Sci..

[94] Kang P.H., Kumar S., Schaffer D.V. (2017). Novel biomaterials to study neural stem cell mechanobiology and improve cell-replacement therapies. Curr. Opin. Biomed. Eng..

[95] Muhamed I., Chowdhury F., Maruthamuthu V. (2017). Biophysical tools to study cellular mechanotransduction. Bioengineering.

[96] Fröhlich E. (2012). The role of surface charge in cellular uptake and cytotoxicity of medical nanoparticles. Int. J. Nanomed..

[97] Salatin S., Maleki Dizaj S., Yari Khosroushahi A. (2015). Effect of the surface modification, size, and shape on cellular uptake of nanoparticles. Cell Biol. Int..

[98] Villanueva-Flores F., Castro-Lugo A., Ramírez O.T., Palomares L.A. (2020). Understanding cellular interactions with nanomaterials: Towards a rational design of medical nanodevices. Nanotechnology.

[99] Yuan T., Gao L., Zhan W., Dini D. (2022). Effect of Particle Size and Surface Charge on Nanoparticles Diffusion in the Brain White Matter. Pharm. Res..

[100] Kalyanasundaram S., Calhoun V.D., Leong K.W. (1997). A finite element model for predicting the distribution of drugs delivered intracranially to the brain. Am. J. Physiol.-Regul. Integr. Comp. Physiol..

[101] Linninger A.A., Somayaji M.R., Mekarski M., Zhang L. (2008). Prediction of convection-enhanced drug delivery to the human brain. J. Theor. Biol..

[102] Somayaji M.R., Xenos M., Zhang L., Mekarski M., Linninger A.A. (2008). Systematic design of drug delivery therapies. Comput. Chem. Eng..

[103] Kim J.H., Astary G.W., Chen X., Mareci T.H., Sarntinoranont M. (2009). Voxelized Model of Interstitial Transport in the Rat Spinal Cord Following Direct Infusion Into White Matter. J. Biomech. Eng..

[104] Kim J.H., Mareci T.H., Sarntinoranont M. (2010). A voxelized model of direct infusion into the corpus callosum and hippocampus of the rat brain: Model development and parameter analysis. Med. Biol. Eng. Comput..

[105] Kim J.H., Astary G.W., Kantorovich S., Mareci T.H., Carney P.R., Sarntinoranont M. (2012). Voxelized Computational Model for Convection-Enhanced Delivery in the Rat Ventral Hippocampus: Comparison with In Vivo MR Experimental Studies. Ann. Biomed. Eng..

[106] Whitaker S. (1986). Flow in porous media I: A theoretical derivation of Darcy’s law. Transp. Porous Media.

[107] Vidotto M., Pederzani M., Castellano A., Pieri V., Falini A., Dini D., De Momi E. (2021). Integrating Diffusion Tensor Imaging and Neurite Orientation Dispersion and Density Imaging to Improve the Predictive Capabilities of CED Models. Ann. Biomed. Eng..

[108] Bernal R., Pullarkat P.A., Melo F. (2007). Mechanical Properties of Axons. Phys. Rev. Lett..

[109] Javid S., Rezaei A., Karami G. (2014). A micromechanical procedure for viscoelastic characterization of the axons and ECM of the brainstem. J. Mech. Behav. Biomed. Mater..

[110] Chen Z.-J., Broaddus W., Viswanathan R., Raghavan R., Gillies G. (2002). Intraparenchymal drug delivery via positive-pressure infusion: Experimental and modeling studies of poroelasticity in brain phantom gels. IEEE Trans. Biomed. Eng..

[111] García J.J., Smith J.H. (2009). A Biphasic Hyperelastic Model for the Analysis of Fluid and Mass Transport in Brain Tissue. Ann. Biomed. Eng..

[112] Smith J.H., García J.J. (2009). A nonlinear biphasic model of flow-controlled infusion in brain: Fluid transport and tissue deformation analyses. J. Biomech..

[113] Smith J.H., Jaime García J. (2011). A nonlinear biphasic model of flow-controlled infusions in brain: Mass transport analyses. J. Biomech..

[114] Ivanchenko O., Sindhwani N., Linninger A. (2010). Experimental Techniques for Studying Poroelasticity in Brain Phantom Gels Under High Flow Microinfusion. J. Biomech. Eng..

[115] Lueshen E., Tangen K., Mehta A.I., Linninger A. (2017). Backflow-free catheters for efficient and safe convection-enhanced delivery of therapeutics. Med. Eng. Phys..

[116] Orozco G.A., Smith J.H., García J.J. (2020). Three-dimensional nonlinear finite element model to estimate backflow during flow-controlled infusions into the brain. Proc. Inst. Mech. Eng. Part H J. Eng. Med..

[117] García J.J., Molano A.B., Smith J.H. (2013). Description and Validation of a Finite Element Model of Backflow During Infusion Into a Brain Tissue Phantom. J. Comput. Nonlinear Dyn..

[118] Stine C.A., Munson J.M. (2019). Convection-Enhanced Delivery: Connection to and Impact of Interstitial Fluid Flow. Front. Oncol..

[119] Linninger A., Tsakiris C., Zhu D., Xenos M., Roycewicz P., Danziger Z., Penn R. (2005). Pulsatile Cerebrospinal Fluid Dynamics in the Human Brain. IEEE Trans. Biomed. Eng..

[120] Linninger A.A., Xenos M., Zhu D.C., Somayaji M.R., Kondapalli S., Penn R.D. (2007). Cerebrospinal Fluid Flow in the Normal and Hydrocephalic Human Brain. IEEE Trans. Biomed. Eng..

[121] Smith J.H., Starkweather K.A., García J.J. (2012). Implications of Transvascular Fluid Exchange in Nonlinear, Biphasic Analyses of Flow-Controlled Infusion in Brain. Bull. Math. Biol..

[122] Wagner A., Ehlers W. (2010). Continuum-Mechanical Analysis of Human Brain Tissue. PAMM.

[123] Wagner A., Ehlers W. (2011). Computational modelling of drug infusion into the anisotropic white-matter tracts of the human brain. PAMM.

[124] Magdoom K.N., Pishko G.L., Rice L., Pampo C., Siemann D.W., Sarntinoranont M. (2014). MRI-Based Computational Model of Heterogeneous Tracer Transport following Local Infusion into a Mouse Hind Limb Tumor. PLoS ONE.

[125] Zhan W., Arifin D.Y., Lee T.K., Wang C.H. (2017). Mathematical Modelling of Convection Enhanced Delivery of Carmustine and Paclitaxel for Brain Tumour Therapy. Pharm. Res..

[126] Zhan W., Wang C.H. (2018). Convection enhanced delivery of liposome encapsulated doxorubicin for brain tumour therapy. J. Control Release.

[127] Zhan W., Wang C.H. (2018). Convection enhanced delivery of chemotherapeutic drugs into brain tumour. J. Control Release.

[128] Zhan W. (2020). Convection enhanced delivery of anti-angiogenic and cytotoxic agents in combination therapy against brain tumour. Eur. J. Pharm. Sci..

[129] Kamali-Zare P., Nicholson C. (2013). Brain extracellular space: Geometry, matrix and physiological importance. Basic Clin. Neurosci..

[130] Nicholson C., Hrabětová S. (2017). Brain Extracellular Space: The Final Frontier of Neuroscience. Biophys. J..

[131] Nicholson C., Kamali-Zare P., Tao L. (2011). Brain extracellular space as a diffusion barrier. Comput. Vis. Sci..

[132] Thorne R.G., Nicholson C. (2006). In vivo diffusion analysis with quantum dots and dextrans predicts the width of brain extracellular space. Proc. Natl. Acad. Sci. USA.

[133] Syková E., Nicholson C. (2008). Diffusion in Brain Extracellular Space. Physiol. Rev..

[134] Tao L., Nicholson C. (2004). Maximum geometrical hindrance to diffusion in brain extracellular space surrounding uniformly spaced convex cells. J. Theor. Biol..

[135] Nicholson C., Kamali-Zare P. (2020). Reduction of Dimensionality in Monte Carlo Simulation of Diffusion in Extracellular Space Surrounding Cubic Cells. Neurochem. Res..

[136] Fieremans E., De Deene Y., Delputte S., Özdemir M.S., D’Asseler Y., Vlassenbroeck J., Deblaere K., Achten E., Lemahieu I. (2008). Simulation and experimental verification of the diffusion in an anisotropic fiber phantom. J. Magn. Reson..

[137] Nilsson M., Lätt J., Ståhlberg F., van Westen D., Hagslätt H. (2012). The importance of axonal undulation in diffusion MR measurements: A Monte Carlo simulation study. NMR Biomed..

[138] Su D., Ma R., Salloum M., Zhu L. (2010). Multi-scale study of nanoparticle transport and deposition in tissues during an injection process. Med. Biol. Eng. Comput..

[139] Su D., Ma R., Zhu L. (2011). Numerical study of nanofluid infusion in deformable tissues for hyperthermia cancer treatments. Med. Biol. Eng. Comput..

[140] Hollingsworth S.A., Dror R.O. (2018). Molecular Dynamics Simulation for All. Neuron.

[141] Dror R.O., Jensen M.Ø., Borhani D.W., Shaw D.E. (2010). Exploring atomic resolution physiology on a femtosecond to millisecond timescale using molecular dynamics simulations. J. Gen. Physiol..

[142] Hoogerbrugge P.J., Koelman J.M.V.A. (1992). Simulating Microscopic Hydrodynamic Phenomena with Dissipative Particle Dynamics. Europhys. Lett. (EPL).

[143] Dai X., Ding H., Yin Q., Wan G., Shi X., Qiao Y. (2015). Dissipative particle dynamics study on self-assembled platycodin structures: The potential biocarriers for drug delivery. J. Mol. Graph. Model..

[144] Sweetman B., Xenos M., Zitella L., Linninger A.A. (2011). Three-dimensional computational prediction of cerebrospinal fluid flow in the human brain. Comput. Biol. Med..

[145] Messaritaki E., Rudrapatna S.U., Parker G.D., Gray W.P., Jones D.K. (2018). Improving the Predictions of Computational Models of Convection-Enhanced Drug Delivery by Accounting for Diffusion Non-gaussianity. Front. Neurol..

[146] Brady M., Raghavan R., Sampson J. (2020). Determinants of Intraparenchymal Infusion Distributions: Modeling and Analyses of Human Glioblastoma Trials. Pharmaceutics.

[147] Bander E.D., Tizi K., Wembacher-Schroeder E., Thomson R., Donzelli M., Vasconcellos E., Souweidane M.M. (2020). Deformational changes after convection-enhanced delivery in the pediatric brainstem. Neurosurg. Focus.

[148] Cheng A.D. (1997). Material coefficients of anisotropic poroelasticity. Int. J. Rock Mech. Min. Sci..

[149] Multiphysics C. (2015). The COMSOL Multiphysics Reference Manual. https://doc.comsol.com/5.5/doc/com.comsol.help.comsol/COMSOL_ReferenceManual.pdf.

[150] Hrabetova S., Cognet L., Rusakov D.A., Nägerl U.V. (2018). Unveiling the extracellular space of the brain: From super-resolved microstructure to in vivo function. J. Neurosci..

[151] Faria C., Erlhagen W., De Momi E., Ferrigno G., Bicho E. (2015). Review of Robotic Technology for Stereotactic Neurosurgery. IEEE Rev. Biomed. Eng..

[152] Smith J.A., Jivraj J., Wong R., Yang V. (2016). 30 Years of Neurosurgical Robots: Review and Trends for Manipulators and Associated Navigational Systems. Ann. Biomed. Eng..

[153] Fomenko A., Serletis D. (2017). Robotic Stereotaxy in Cranial Neurosurgery: A Qualitative Systematic Review. Neurosurgery.

[154] Wang M.Y., Goto T., Tessitore E., Veeravagu A. (2017). Introduction. Robotics in neurosurgery. Neurosurg. Focus FOC.

[155] Kwoh Y.S., Hou J., Jonckheere E.A., Hayati S. (1988). A robot with improved absolute positioning accuracy for CT guided stereotactic brain surgery. IEEE Trans. Biomed. Eng..

[156] Drake J.M., Joy M., Goldenberg A., Kreindler D. (1991). Computer- and robot-assisted resection of thalamic astrocytomas in children. Neurosurgery.

[157] Li Q.H., Zamorano L., Pandya A., Perez R., Gong J., Diaz F. (2002). The application accuracy of the NeuroMate robot—A quantitative comparison with frameless and frame-based surgical localization systems. Comput. Aided Surg..

[158] Lewis O., Woolley M., Johnson D., Rosser A., Barua N.U., Bienemann A.S., Gill S.S., Evans S. (2016). Chronic, intermittent convection-enhanced delivery devices. J. Neurosci. Methods.

[159] van de Berg N.J., van Gerwen D.J., Dankelman J., van den Dobbelsteen J.J. (2015). Design Choices in Needle Steering—A Review. IEEE/ASME Trans. Mechatron..

[160] EDEN2020 Enhanced Delivery Ecosystem for Neurosurgery in 2020. https://www.eden2020.eu..

[161] Blumenfeld P., Hata N., DiMaio S., Zou K., Haker S., Fichtinger G., Tempany C.M. (2007). Transperineal prostate biopsy under magnetic resonance image guidance: A needle placement accuracy study. J. Magn. Reson. Imaging Off. J. Int. Soc. Magn. Reson. Med..

[162] Schouten M.G., Bomers J.G., Yakar D., Huisman H., Rothgang E., Bosboom D., Scheenen T.W., Misra S., Fütterer J.J. (2012). Evaluation of a robotic technique for transrectal MRI-guided prostate biopsies. Eur. Radiol..

[163] Patil S., Burgner J., Webster R.J., Alterovitz R. (2014). Needle Steering in 3-D Via Rapid Replanning. IEEE Trans. Robot..

[164] Essert C., Haegelen C., Lalys F., Abadie A., Jannin P. (2012). Automatic computation of electrode trajectories for deep brain stimulation: A hybrid symbolic and numerical approach. Int. J. Comput. Assist. Radiol. Surg..

[165] Segato A., Sestini L., Castellano A., De Momi E. GA3C Reinforcement Learning for Surgical Steerable Catheter Path Planning. Proceedings of the 2020 IEEE International Conference on Robotics and Automation (ICRA).

[166] Favaro A., Segato A., Muretti F., Momi E.D. (2021). An Evolutionary-Optimized Surgical Path Planner for a Programmable Bevel-Tip Needle. IEEE Trans. Robot..

[167] Likhachev M., Ferguson D., Gordon G., Stentz A., Thrun S. Anytime Dynamic A*: An Anytime, Replanning Algorithm. Proceedings of the Fifteenth International Conference on International Conference on Automated Planning and Scheduling (ICAPS’05).

[168] Leibrandt K., Bergeles C., Yang G.Z. (2017). Concentric Tube Robots: Rapid, Stable Path-Planning and Guidance for Surgical Use. IEEE Robot. Autom. Mag..

[169] Patil S., Alterovitz R. Interactive motion planning for steerable needles in 3D environments with obstacles. Proceedings of the 2010 3rd IEEE RAS EMBS International Conference on Biomedical Robotics and Biomechatronics.

[170] Fauser J., Sakas G., Mukhopadhyay A. (2018). Planning nonlinear access paths for temporal bone surgery. Int. J. Comput. Assist. Radiol. Surg..

[171] Yang K., Sukkarieh S. 3D smooth path planning for a UAV in cluttered natural environments. Proceedings of the 2008 IEEE/RSJ International Conference on Intelligent Robots and Systems.

[172] Favaro A., Cerri L., Galvan S., Baena F.R.Y., De Momi E. Automatic Optimized 3D Path Planner for Steerable Catheters with Heuristic Search and Uncertainty Tolerance. Proceedings of the 2018 IEEE International Conference on Robotics and Automation (ICRA).

[173] Liu F., Garriga-Casanovas A., Secoli R., Rodriguez y Baena F. (2016). Fast and Adaptive Fractal Tree-Based Path Planning for Programmable Bevel Tip Steerable Needles. IEEE Robot. Autom. Lett..

[174] Pinzi M., Galvan S., Rodriguez y Baena F. (2019). The Adaptive Hermite Fractal Tree (AHFT): A novel surgical 3D path planning approach with curvature and heading constraints. Int. J. Comput. Assist. Radiol. Surg..

[175] Fu M., Salzman O., Alterovitz R. (2021). Toward Certifiable Motion Planning for Medical Steerable Needles. Proceedings of Robotics: Science and Systems. arXiv.

[176] Barraquand J., Latombe J.C. Nonholonomic multibody mobile robots: Controllability and motion planning in the presence of obstacles. Proceedings of the Proceedings. 1991 IEEE International Conference on Robotics and Automation.

[177] Lindemann S., LaValle S. Multiresolution approach for motion planning under differential constraints. Proceedings of the 2006 IEEE International Conference on Robotics and Automation, 2006. ICRA 2006.

[178] Pinzi M., Watts T., Secoli R., Galvan S., Baena F.R.y. (2021). Path Replanning for Orientation-Constrained Needle Steering. IEEE Trans. Biomed. Eng..

[179] Quinlan S., Khatib O. Elastic bands: Connecting path planning and control. Proceedings of the [1993] Proceedings IEEE International Conference on Robotics and Automation.

[180] Lee C.T., Tsai C.C., Li T.H.S., Tu K.Y., Tsai C.C., Hsu C.C., Tseng C.C., Vadakkepat P., Baltes J., Anderson J., Wong C.C., Jesse N. (2011). 3D Collision-Free Trajectory Generation Using Elastic Band Technique for an Autonomous Helicopter. Next Wave in Robotics.

[181] Zhu Z., Schmerling E., Pavone M. A convex optimization approach to smooth trajectories for motion planning with car-like robots. Proceedings of the 2015 54th IEEE Conference on Decision and Control (CDC).

[182] Brainlab AG Cranial Navigation Application. https://www.brainlab.com/surgery-products/overview-neurosurgery-products/cranial-navigation/.

[183] Medtronic plc.Stealth Navigation for Neurosurgery. https://www.medtronic.com/us-en/healthcare-professionals/products/neurological/surgical-navigation-systems.html.

[184] Renishaw plc.Neuroinspire. https://www.renishaw.com/en/neuroinspire-neurosurgical-planning-software–8244.

[185] Chittiboina P., Heiss J.D., Lonser R.R. (2015). Accuracy of direct magnetic resonance imaging-guided placement of drug infusion cannulae. J. Neurosurg..

[186] Chen Y., Godage I.S., Sengupta S., Liu C.L., Weaver K.D., Barth E.J. (2019). MR-conditional steerable needle robot for intracerebral hemorrhage removal. Int. J. Comput. Assist. Radiol. Surg..

[187] Patel N.A., van Katwijk T., Li G., Moreira P., Shang W., Misra S., Fischer G.S. Closed-loop asymmetric-tip needle steering under continuous intraoperative MRI guidance. Proceedings of the 2015 37th Annual International Conference of the IEEE Engineering in Medicine and Biology Society (EMBC).

[188] Matheson E., Rodriguez y Baena F. (2020). Biologically Inspired Surgical Needle Steering: Technology and Application of the Programmable Bevel-Tip Needle. Biomimetics.

[189] Boviatsis E.J., Kouyialis A.T., Stranjalis G., Korfias S., Sakas D.E. (2003). CT-guided stereotactic aspiration of brain abscesses. Neurosurg. Rev..

[190] Bhattacharji P., Moore W. (2017). Application of real-time 3D navigation system in CT-guided percutaneous interventional procedures: A feasibility study. Radiol. Res. Pract..

[191] Lo Presti D., Massaroni C., Jorge Leitão C.S., De Fátima Domingues M., Sypabekova M., Barrera D., Floris I., Massari L., Oddo C.M., Sales S. (2020). Fiber Bragg Gratings for Medical Applications and Future Challenges: A Review. IEEE Access.

[192] Chevrie J., Shahriari N., Babel M., Krupa A., Misra S. (2018). Flexible Needle Steering in Moving Biological Tissue With Motion Compensation Using Ultrasound and Force Feedback. IEEE Robot. Autom. Lett..

[193] Khan F., Denasi A., Barrera D., Madrigal J., Sales S., Misra S. (2019). Multi-Core Optical Fibers With Bragg Gratings as Shape Sensor for Flexible Medical Instruments. IEEE Sens. J..

[194] Khan F., Donder A., Galvan S., Baena F.R.y., Misra S. (2020). Pose Measurement of Flexible Medical Instruments Using Fiber Bragg Gratings in Multi-Core Fiber. IEEE Sens. J..

[195] Brainlab AG Intraoperative Ultrasound. https://www.brainlab.com/surgery-products/overview-neurosurgery-products/intraoperative-ultrasound/.

[196] Scholten H., Pourtaherian A., Mihajlovic N., Korsten H., A. Bouwman R. (2017). Improving needle tip identification during ultrasound-guided procedures in anaesthetic practice. Anaesthesia.

[197] Denasi A., Khan F., Boskma K.J., Kaya M., Hennersperger C., Göbl R., Tirindelli M., Navab N., Misra S. An Observer-Based Fusion Method Using Multicore Optical Shape Sensors and Ultrasound Images for Magnetically-Actuated Catheters. Proceedings of the 2018 IEEE International Conference on Robotics and Automation (ICRA).

[198] Gerard I.J., Kersten-Oertel M., Petrecca K., Sirhan D., Hall J.A., Collins D.L. (2017). Brain shift in neuronavigation of brain tumors: A review. Med. Image Anal..

[199] Fuerst B., Wein W., Müller M., Navab N. (2014). Automatic ultrasound–MRI registration for neurosurgery using the 2D and 3D LC2 Metric. Med. Image Anal..

[200] Drobny D., Ranzini M., Ourselin S., Vercauteren T., Modat M., Zhou L., Heller N., Shi Y., Xiao Y., Sznitman R., Cheplygina V., Mateus D., Trucco E., Hu X.S., Chen D. (2019). Landmark-Based Evaluation of a Block-Matching Registration Framework on the RESECT Pre- and Intra-operative Brain Image Data Set. Large-Scale Annotation of Biomedical Data and Expert Label Synthesis and Hardware Aware Learning for Medical Imaging and Computer Assisted Intervention.

[201] Heinrich M.P., Jenkinson M., Papież B.W., Brady M., Schnabel J.A. (2013). Towards realtime multimodal fusion for image-guided interventions using self-similarities. International Conference on Medical Image Computing and Computer-Assisted Intervention.

[202] Göbl R., Navab N., Hennersperger C. (2018). SUPRA: Open-source software-defined ultrasound processing for real-time applications. Int. J. Comput. Assist. Radiol. Surg..

[203] Forte A.E., Galvan S., Dini D. (2018). Models and tissue mimics for brain shift simulations. Biomech. Model. Mechanobiol..

[204] Leibinger A., Forte A.E., Tan Z., Oldfield M.J., Beyrau F., Dini D., Rodriguez y Baena F. (2016). Soft Tissue Phantoms for Realistic Needle Insertion: A Comparative Study. Ann. Biomed. Eng..

[205] Tan Z., Dini D., Rodriguez y Baena F., Forte A.E. (2018). Composite hydrogel: A high fidelity soft tissue mimic for surgery. Mater. Des..

[206] Budday S., Sommer G., Birkl C., Langkammer C., Haybaeck J., Kohnert J., Bauer M., Paulsen F., Steinmann P., Kuhl E. (2017). Mechanical characterization of human brain tissue. Acta Biomater..

[207] Comellas E., Budday S., Pelteret J.P., Holzapfel G.A., Steinmann P. (2020). Modeling the porous and viscous responses of human brain tissue behavior. Comput. Methods Appl. Mech. Eng..

[208] Rasin I., Pekar Z., Sadowsky O., Forte A., Galvan S., Dini D., Shoham M., Joskowicz L. Real-time modeling of intra-operative brain shift based on video tracking. Proceedings of the Hamlyn Symposium on Medical Robotics.

[209] Dumpuri P., Thompson R.C., Dawant B.M., Cao A., Miga M.I. (2007). An atlas-based method to compensate for brain shift: Preliminary results. Med. Image Anal..

[210] BWH 3D Slicer. https://www.slicer.org/.

[211] DFKZ The Medical Imaging Interaction Toolkit (MITK). https://www.mitk.org.

[212] Tokuda J., Fischer G.S., Papademetris X., Yaniv Z., Ibanez L., Cheng P., Liu H., Blevins J., Arata J., Golby A.J. (2009). OpenIGTLink: An open network protocol for image-guided therapy environment. Int. J. Med. Robot. Comput. Assist. Surg..

[213] SOFA Simulation Open Framework Architecture. https://www.sofa-framework.org.

[214] Kitware Interactive Medical Simulation Toolkit. https://www.imstk.org.

[215] Mueller S., Polley M.Y., Lee B., Kunwar S., Pedain C., Wembacher-Schröder E., Mittermeyer S., Westphal M., Sampson J.H., Vogelbaum M.A. (2011). Effect of imaging and catheter characteristics on clinical outcome for patients in the PRECISE study. J. Neuro-Oncol..

[216] Matheson E., Secoli R., Galvan S., Rodriguez y Baena F. Human-robot visual interface for 3D steering of a flexible, bioinspired needle for neurosurgery. Proceedings of the 2019 IEEE/RSJ International Conference on Intelligent Robots and Systems (IROS).

[217] Mulder M., Mulder J., Stassen H. Cybernetics of tunnel-in-the-sky displays. II. Curved trajectories. Proceedings of the IEEE SMC’99 Conference Proceedings. 1999 IEEE International Conference on Systems, Man, and Cybernetics (Cat. No. 99CH37028).

[218] Yuan C., Recktenwald F., Mallot H.A. Visual steering of UAV in unknown environments. Proceedings of the 2009 IEEE/RSJ International Conference on Intelligent Robots and Systems.

[219] Castellano A., Falini A. (2016). Progress in neuro-imaging of brain tumors. Curr. Opin. Oncol..

[220] Riva M., Lopci E., Gay L.G., Nibali M.C., Rossi M., Sciortino T., Castellano A., Bello L. (2021). Advancing Imaging to Enhance Surgery: From Image to Information Guidance. Neurosurg. Clin. N. Am..

[221] Jahangiri A., Chin A.T., Flanigan P.M., Chen R., Bankiewicz K., Aghi M.K. (2017). Convection-enhanced delivery in glioblastoma: A review of preclinical and clinical studies. J. Neurosurg..

[222] Lonser R.R. (2017). Imaging of Convective Drug Delivery in the Nervous System. Neurosurg. Clin. N. Am..

[223] Castellano A., Cirillo S., Bello L., Riva M., Falini A. (2017). Functional MRI for Surgery of Gliomas. Curr. Treat. Opt. Neurol..

[224] Pieri V., Sanvito F., Riva M., Petrini A., Rancoita P.M.V., Cirillo S., Iadanza A., Bello L., Castellano A., Falini A. (2021). Along-tract statistics of neurite orientation dispersion and density imaging diffusion metrics to enhance MR tractography quantitative analysis in healthy controls and in patients with brain tumors. Hum. Brain Mapp..

[225] Castellano A., Bello L., Michelozzi C., Gallucci M., Fava E., Iadanza A., Riva M., Casaceli G., Falini A. (2012). Role of diffusion tensor magnetic resonance tractography in predicting the extent of resection in glioma surgery. Neuro Oncol..

[226] Altabella L., Broggi S., Mangili P., Conte G.M., Pieri V., Iadanza A., Del Vecchio A., Anzalone N., di Muzio N., Calandrino R. (2018). Integration of Diffusion Magnetic Resonance Tractography into tomotherapy radiation treatment planning for high-grade gliomas. Phys. Med..

[227] Segato A., Pieri V., Favaro A., Riva M., Falini A., De Momi E., Castellano A. (2019). Automated Steerable Path Planning for Deep Brain Stimulation Safeguarding Fiber Tracts and Deep Gray Matter Nuclei. Front. Robot. AI.

[228] D’Amico R.S., Neira J.A., Yun J., Alexiades N.G., Banu M., Englander Z.K., Kennedy B.C., Ung T.H., Rothrock R.J., Romanov A. (2019). Validation of an effective implantable pump-infusion system for chronic convection-enhanced delivery of intracerebral topotecan in a large animal model. J. Neurosurg..

[229] Gimenez F., Krauze M.T., Valles F., Hadaczek P., Bringas J., Sharma N., Forsayeth J., Bankiewicz K.S. (2011). Image-guided convection-enhanced delivery of GDNF protein into monkey putamen. NeuroImage.

[230] van der Bom I.M., Moser R.P., Gao G., Sena-Esteves M., Aronin N., Gounis M.J. (2013). Frameless multimodal image guidance of localized convection-enhanced delivery of therapeutics in the brain. J. Neurointerv. Surg..

[231] Sewing A.C.P., Lagerweij T., van Vuurden D.G., Meel M.H., Veringa S.J.E., Carcaboso A.M., Gaillard P.J., Peter Vandertop W., Wesseling P., Noske D. (2017). Preclinical evaluation of convection-enhanced delivery of liposomal doxorubicin to treat pediatric diffuse intrinsic pontine glioma and thalamic high-grade glioma. J. Neurosurg. Pediatr..

[232] Tromp D.P., Adluru N., Alexander A.L., Emborg M.E. (2011). Simulating convection-enhanced delivery in the putamen using probabilistic tractography. Proc. IEEE Int. Symp. Biomed. Imaging.

[233] Rosenbluth K.H., Martin A.J., Bringas J., Bankiewicz K.S. (2012). Evaluation of pressure-driven brain infusions in nonhuman primates by intra-operative 7 Tesla MRI. J. Magn. Reson. Imaging.

[234] Raghavan R., Brady M.L., Rodríguez-Ponce M.I., Hartlep A., Pedain C., Sampson J.H. (2006). Convection-enhanced delivery of therapeutics for brain disease, and its optimization. Neurosurg. Focus.

[235] Arifin D.Y., Lee K.Y., Wang C.H., Smith K.A. (2009). Role of convective flow in carmustine delivery to a brain tumor. Pharm. Res..

[236] Banks J. (2015). The Neurotechnological Revolution: Unlocking the brain’s secrets to develop innovative technologies as well as treatments for neurological diseases. IEEE Pulse.

[237] Murray C.J., Lopez A.D. (1997). Global mortality, disability, and the contribution of risk factors: Global Burden of Disease Study. Lancet.

[238] DiLuca M., Olesen J. (2014). The Cost of Brain Diseases: A Burden or a Challenge?. Neuron.

[239] Sobocki P., Lekander I., Berwick S., Olesen J., Jönsson B. (2006). Resource allocation to brain research in Europe (RABRE). Eur. J. Neurosci..

[240] Kesari S. (2011). Understanding Glioblastoma Tumor Biology: The Potential to Improve Current Diagnosis and Treatments. Semin. Oncol..

[241] Portnow J., Badie B., Chen M., Liu A., Blanchard S., Synold T.W. (2009). The Neuropharmacokinetics of Temozolomide in Patients with Resectable Brain Tumors: Potential Implications for the Current Approach to Chemoradiation. Clin. Cancer Res..

[242] Ostermann S., Csajka C., Buclin T., Leyvraz S., Lejeune F., Decosterd L.A., Stupp R. (2004). Plasma and Cerebrospinal Fluid Population Pharmacokinetics of Temozolomide in Malignant Glioma Patients. Clin. Cancer Res..

